# Progress in Modeling and Applications of Solid Electrolyte Interphase Layers for Lithium Metal Anodes

**DOI:** 10.3390/nano15070554

**Published:** 2025-04-05

**Authors:** Zhicong Wei, Weitao Zheng, Yijuan Li, Shaoming Huang

**Affiliations:** 1 Guangzhou Key Laboratory of Low-Dimensional Materials and Energy Storage Devices, Collaborative Innovation Center of Advanced Energy Materials, School of Materials and Energy, Guangdong University of Technology, Guangzhou 510006, China; 3122005322@mail2.gdut.edu.cn (Z.W.); 2112202253@mail2.gdut.edu.cn (W.Z.); 2 School of Chemistry and Materials Science, Hangzhou Institute for Advanced Study, University of Chinese Academy of Sciences, Hangzhou 310024, China

**Keywords:** solid electrolyte interphase layer, mathematical model, SEI construction

## Abstract

The increasing demand for high-specific-energy lithium batteries has stimulated extensive research on the lithium metal anode owing to its high specific capacity and low electrode potential. However, the lithium metal will irreversibly react with the electrolyte during the first cycling process, forming an uneven and unstable solid electrolyte interphase (SEI) layer, which results in the non-uniform deposition of Li ions and thus the formation of lithium dendrites. This could cause a battery short circuit, resulting in safety hazards such as thermal runaway. In addition, the continuous rupture and repair of the SEIs during the repeated charge/discharge processes will constantly consume the active lithium, which leads to a significant decrease in battery capacity. An effective strategy to address these challenges is to design and construct ideal artificial SEIs on the surface of the lithium metal anode. This review analyzes and summarizes the mathematical modeling of SEI, the functional characteristics of SEIs with different components, and finally discusses the challenges faced by artificial SEIs in practical applications of lithium metal batteries and future development directions.

## 1. Introduction

In recent years, the market demand for vehicle electrification, equipment automation, and renewable energy harvesting has increased exponentially, significantly promoting the research of high-energy-density batteries. Lithium metal batteries (LMBs), especially lithium–sulfur batteries and lithium–oxygen batteries, have been considered as one of the most promising high-capacity battery systems because the lithium metal anode (LMA) has an ultra-high theoretical capacity (3860 mAh g^−1^) and low electrochemical potential (−3.04 V vs. standard hydrogen electrode) [1,2,3]. However, the realization of LMAs still faces a series of challenges, including the uncontrolled growth of lithium dendrites [4,5,6], the formation of “dead lithium” [7,8,9], infinite volume changes [10,11,12], and unstable SEI [13,14,15]. When in contact with the electrolyte, the lithium metal will spontaneously react with it, gradually forming an intrinsic SEI on the lithium surface. Unfortunately, this natural SEI is unstable and easily destroyed during the cycle processes [16]. In addition, the lithium ions are more inclined to gather in the SEI cracks, resulting in the uneven deposition of Li ions and thus the formation of lithium dendrites, which may cause short circuits in the batteries. At the same time, the rupture and repair process of SEI requires continuous consumption of active lithium and electrolytes, resulting in rapid capacity decay and final cell failure [17]. Moreover, the side reaction of lithium metal with moisture and other gases in the air poses another challenge, leading to the formation of many unpredictable multi-component oxide layers on the surface of lithium. To cope with this challenge, the assembly process of LMBs requires an absolutely water-free and oxygen-free environment, thus increasing the cost of battery assembly in the large-scale manufacturing process [18,19,20]. In view of the above problems, the most feasible strategy is to actively construct an artificial protective layer on the surface of lithium metal to inhibit the dendrites growth and reduce the side reactions with electrolyte or gases in the air.

Some companies have made significant progress in the solution of stabilizing the SEI in liquid electrolytes. For example, Prologium Technology demonstrated its fourth-generation lithium ceramic battery system at the Consumer Electronics Show (CES) 2025, which uses all-inorganic electrolyte technology to completely eliminate organic components and increase inorganic components from 90% to 100%. This technology not only improves the energy density, charging speed, and low-temperature reliability of the battery but also significantly enhances the safety. In addition, SES AI Corporation has also successfully achieved a stable SEI in liquid electrolyte through its hybrid lithium metal battery technology. The hybrid lithium metal battery of SES performs well in terms of energy density and cycle life. The technology maintains the use of liquid electrolytes by introducing lithium metal anodes into the traditional lithium-ion battery structure. Through innovative technologies and materials, these companies have provided viable solutions for the commercial application of lithium metal batteries and promoted the development of the entire industry.

As early as 1979, the term “solid electrolyte interphase” was introduced by Peled [21] to describe the protective layer that impedes the ongoing interactions between the non-aqueous electrolyte and the alkali metal anode. In addition, the term “solid electrolyte” is employed to describe both its physical state and functionality, as the film exhibits characteristics akin to those of a solid-state electrolyte, including ionic conductivity and electronic insulation. The introduction of the term “interphase” brought forth a significant concept, indicating that this film exists as an autonomous phase, which serves as an intermediary between the electrolyte and lithium metal. Subsequently, the term “SEI” was widely adopted to denote analogous interphases formed in diverse anode materials, such as graphite, silicon (Si), lithium, etc. [22]. This paper reviews the modeling progress, application research, existing challenges, and future development directions of SEIs in lithium metal batteries.

## 2. SEI Formation Mechanism

**Figure 1:**
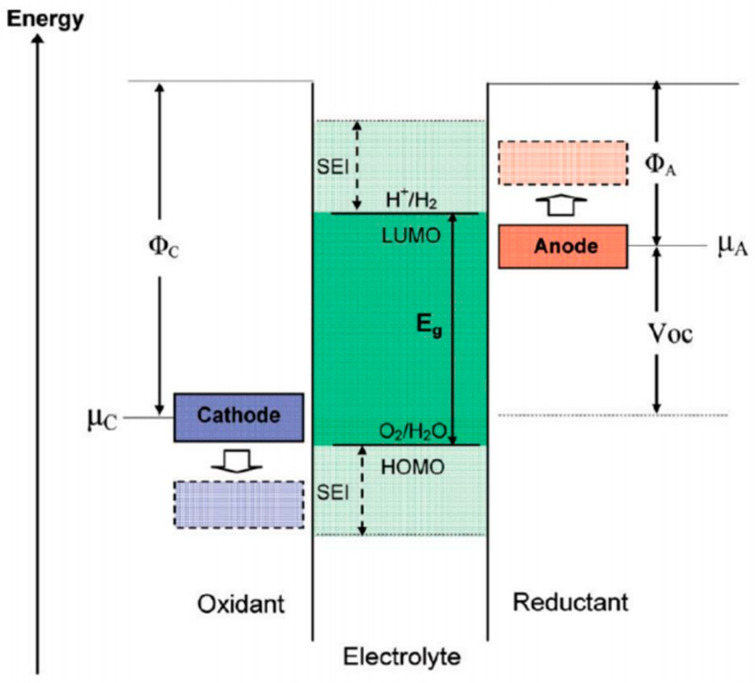
Energy diagram of open circuit potential of electrolyte, where Φ_A_ and Φ_C_ are the conduction functions of the anode and cathode in the circuit. E_g_ is the band gap. μ_A_ and μ_C_ are the REDOX potentials of the anode and cathode, respectively [23].

The formation mechanism of SEIs in LMBs is a complex multi-step process involving the interaction between the electrode surface and electrolyte. In the production and manufacture of batteries, the SEI is naturally formed during the initial charging and discharging process without artificial induction. Specifically, the solvent, lithium salts, and additives in the electrolyte are reduced on the lithium anode surface to form an SEI in LMBs. These reactions usually involve both the organic matter in the electrolyte and active lithium metal, forming a protective layer to prevent further side reactions. The reduction reaction for each electrolyte component varies based on its lowest unoccupied molecular orbital (LUMO) below the Fermi level of the anode, as proposed by Goodenough [23] (Figure 1). If the LUMO of the electrolyte is lower than the Fermi level of lithium, the electrons will be transferred to the electrolyte, resulting in a reduction reaction of the electrolyte molecules to decompose into products such as lithium salts (LiF, Li_2_CO_3_, etc.) and organic compounds, which accumulate on the surface of the lithium metal anode to form a SEI. It is important to note that this explanation solely focuses on the chemical reactions associated with the formation of SEIs, while the actual battery internal reactions involve complex conditions influenced by factors such as voltage, temperature, pressure, and so on [24].

After understanding the underlying chemical reaction mechanism behind the formation of SEIs, the next step is to study the specific formation process of SEIs during cycling and their impact on the battery performance. The formation process of the SEI on the lithium metal anode usually occurs during the first charge–discharge process of the battery [25]. When the battery starts to work, the surface of the lithium metal anode is in contact with the electrolyte. Under the action of voltage, the solvent and salt in the electrolyte undergo a reduction reaction. Solvents in the electrolyte, such as ethylene carbonate (EC) or dimethyl carbonate (DMC), are reduced on the surface of lithium metal and decomposed into lithium salts (such as LiF, Li_2_CO_3_) and organic compounds. These products form a thin and dense solid layer covering the surface of lithium metal, which plays a protective role. The formation of the SEI can prevent the electrolyte from further reacting with the lithium metal, thereby reducing side reactions and corrosion of the lithium metal. In addition, the SEI can effectively isolate the direct contact between the lithium metal and the electrolyte, slow down the decomposition of the electrolyte, and maintain the stability and long life of the battery. It is challenging to precisely determine the thickness of SEIs because they exist in the electrolyte solutions. However, scientists have found that it is possible to provide an approximate estimation of the average thickness of SEI between the active substance and electrolyte via ionic impedance technology, which ranges from nanometers to hundreds of nanometers [26]. This layer acts as an insulator for electrons while providing a highly conductive pathway for lithium ions [27], which brings both advantages and disadvantages. On one hand, a dense and complete SEI can significantly weaken electron tunneling, thereby preventing the further depletion of electrolytes and protecting the lithium anode from further damage caused by solvent molecules. On the other hand, the formation and growth of SEIs consume active lithium as well as the materials present in the electrolyte solution, resulting in a series of problems such as increased battery impedance, a decrease in coulombic efficiency, and the attenuation of battery capacity [28,29]. At the same time, the uneven deposition of Li^+^ in the cracks of SEI leads to the formation of lithium dendrites, which may puncture the separator and cause a short circuit in the batteries, resulting in safety hazards such as thermal runaway. Consequently, extensive research has been conducted to understand the formation mechanism, stability, and influencing factors of the SEI [30,31], with the aim of improving the battery performance. Indeed, the electrode–electrolyte interphase layers exist not only on the anodes but also on the cathode. The layer formed on the surface of the positive electrode is known as the cathode–electrolyte interphase (CEI) layer, which also increases the interphase impedance and slightly reduces the reversible cycle capacity [32]. This review focuses on the SEIs on the surface of lithium metal anodes.

## 3. Overview of SEI Modeling Development

Researchers have experienced a long process of understanding the SEIs because of their complex compositions. The understanding of this protective layer on the negative electrodes originated from its observation on lithium metal immersed in a non-aqueous electrolyte in 1979 by Peled [21]. In the same year, Peled introduced the concept of SEI, which is a protective layer of electronic insulation while forming an ion conduction between the electrode and the electrolyte, playing a similar role to a solid electrolyte. Over two decades of observation and summary, the model was fully enriched and improved by Peled [33] in 1997 and by Aurbach et al. [25] in 1999. Even today, the SEI remains “the most important but least understood component” in rechargeable LMBs because of the complex chemical reactions that occur during its formation process and inadequate measurement of their physical properties [26].

Owing to the intricate reaction between the electrolyte and the electrode surface, there is no comprehensive and systematic theory for the formation and growth of SEIs. The extremely short time scale of the electrochemical reactions associated with SEI makes it challenging to experimentally observe its formation. Without the experimental data as a basis, the difficulty of mathematical theoretical modeling is greatly increased, and it will be more difficult to uncover the secrets of the SEIs. However, some experimental techniques such as Fourier transform infrared spectrometer (FTIR) [34], X-ray photoelectron spectroscopy (XPS) [35], transmission electron microscopy (TEM) [36], and isotope labeling are developing rapidly, which can be employed to investigate the structure and composition of the SEIs. Additionally, the electrochemical quartz crystal microbalance (EQCM) [37] and time-of-flight secondary ion mass spectrometry (ToF-SIMS) [38] are powerful techniques for studying the formation, composition, and properties of SEIs. The EQCM detects the mass changes and mechanical properties of the SEIs by monitoring frequency changes in the electrode coating, while the ToF-SIMS utilizes chemical analysis with high spatial resolution to reveal the detailed composition and structure of the SEIs. The combined use of these two techniques can provide a comprehensive understanding of the characteristics of the SEIs, which can help optimize the electrochemical performances of batteries. With the development of computer technology, the theoretical calculations such as density functional theory (DFT) [34,39], molecular dynamics (MD) [39], and phase-field models (PFM) [40] have been increasingly utilized in SEI research. Therefore, there are two main approaches to studying the SEI: the experimental methods primarily focus on exploring its structure and detecting its composition, while the theoretical calculations are more inclined towards investigating the initial formation mechanism of the SEI and predicting its properties.

### 3.1. Development of Composition and Structure Model for SEI

As mentioned in Section 2, the composition of SEI is complex, involving the occurrence of multiple side reactions and the participation of many uncertain components. The reduction reaction of the electrolyte generates various products, including inorganic salts, organic substances, and gases. These solid byproducts gradually contribute to the growth of SEI during battery operation. Therefore, it is difficult but also very important to study the components of SEI and establish a model that can reflect its components.

The formation and composition of SEI have been extensively investigated in recent decades because of its ability to identify the factors that affect the SEI and provide valuable insights for improving battery performance. However, it is challenging to observe the continuous evolution of the SEI formation and growth under experimental conditions due to the complexity of battery structure and reactions. Consequently, theoretical calculations such as DFT and MD play a significant role in understanding the development of SEI. However, for the growth of lithium dendrites, to further study such problems and provide theoretical guidance for the construction of the SEI, the phase-field modeling (PFM) is a part that cannot be ignored. In recent years, there have been many frontier reports [41,42,43] in this direction, which have made significant contributions to solving the problem of lithium dendrite growth.

**Figure 2:**
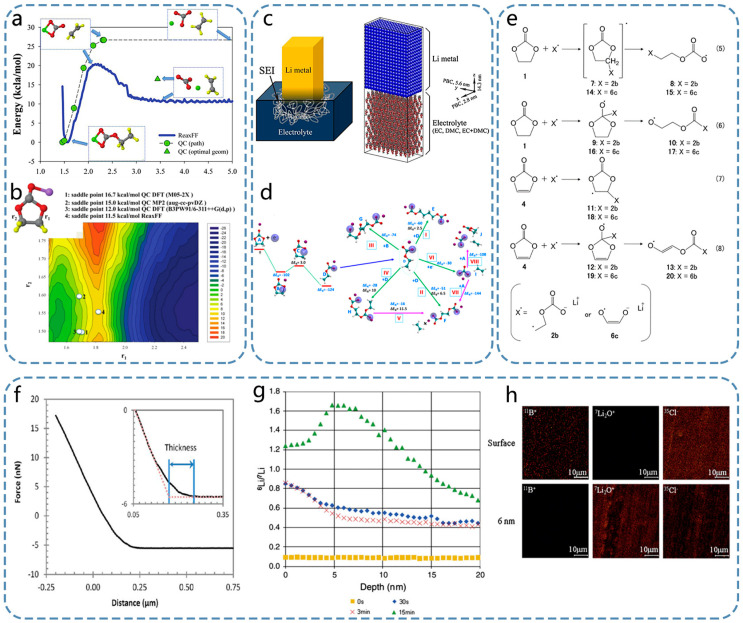
Schematic diagram of theoretical calculation, simulation, and experimental analysis. (**a**) The energy profile for the dissociation of C_2_H_4_ from o-EC^−^/Li^+^ was determined through QC calculations and gas phase simulations using ReaxFF at 10 K. (**b**) ReaxFF simulations at 10 K were utilized to generate a contour map illustrating the opening of R_1_ and R_2_ in c-EC^−^/Li^+^ [48]. (**c**) Illustration of an electrolyte-immersed lithium metal electrode and the initial setup of the cell employed in the simulations 51. (**d**) Energy profile of EC/Li^+^ reduction and radical termination reactions via different pathways, with ΔE_R_ representing reaction energy and ΔE_B_ indicating reaction barrier. Color coding: cyan for carbon, white for hydrogen, red for oxygen, purple for Li^+^, and a large blue sphere symbolizing an electron 50. (**e**) Potential interactions between intact EC (1) and VC (4) molecules with the decomposed anion radicals of EC (2) and VC (6b) [53]. (**f**) An illustration of the force-distance approach curve exhibited by an AFM probe towards a SEI formed in the control system is presented. The inset provides a magnified view for determining thickness, with the same axes as the larger graph. Dotted lines depict the behavior of the probe on an SEI-free surface in electrolyte prior to SEI formation, indicating its response on a solid surface [54]. (**g**) Revealing the SIMS depth profile of the isotope ratio between ^6^Li^+^ and ^7^Li^+^ in the SEI formed by immersing ^7^LiClO_4_ in a ^6^LiBF_4_ electrolyte for different durations: 30 s, 3 min, and 15 min. (**h**) SIMS maps were generated to visualize the distribution of 11B^+^, ^7^Li_2_O^+^, and Cl^−^ ions on both the surface and at a depth of 6 nm below the SEI. The measurements were conducted on a ^7^LiClO_4_ SEI sample immersed in a ^6^LiBF_4_ electrolyte for a duration of 3 min. Areas with higher ion signals are represented by brighter regions [63].

Takenaka conducted simulations by using a combination of Monte Carlo (MC) and molecular dynamics techniques to investigate the reduction process of electrolytes, particularly ethylene carbonate (EC) and propylene carbonate (PC) [41,42]. The layer adjacent to the graphite anode is mainly composed of inorganic compounds, such as Li_2_CO_3_ and LiF. Instead, the outer layer consists of organic substances, such as lithium ethyl carbonate (LiEC), dilithium butylene dicarbonate (Li_2_BDC), or lithium phthalocyanine (LiPC). The SEI formed by EC reduction had a denser structure than that formed by PC reduction. Traditional force fields used in MD simulations, including the Condensed-phase Optimized Molecular Potentials for Atomistic Simulation Studies (COMPASS) [43] and universal force field (UFF) [44] models, have some limitations because they cannot accurately simulate the formation and breakage of chemical bonds during the reaction processes. However, with the introduction of the Reactive Force Field (ReaxFF) reaction force field [45,46,47], it is now possible to more realistically simulate these chemical transformations within MD simulations by considering the interactions between molecules and atoms. Bedrov [48] studied the EC reduction reactions by using the ReaxFF force field and identified the presence of (CH_2_OCO_2_Li)_2_ as one of the products (Figure 2a,b). By analyzing the energy curve (Figure 2a), the key steps in the reaction process, such as adsorption, dissociation, and diffusion, can be identified. This is of great significance for understanding the reaction mechanism and optimizing the reaction conditions. At the same time, the contour map (Figure 2b) clearly shows the energy changes at each position in the R1 and R2 opening process in c-EC^−^/Li^+^, which helps to determine the energy barrier and reaction heat of the reaction so as to understand the difficulty and thermodynamic stability of the reaction. Subsequent studies have also employed this method to investigate EC reduction reactions with similar results [49,50] (Figure 2c,d). Figure 2c is the test device they studied, and Figure 2d is the reaction process they tested out, as well as the barrier information of each step. The ReaxFF force field provides a detailed understanding of EC reduction reactions at the atomic level, allowing researchers to identify specific products. Although it works well in controlled simulations, the ReaxFF force field may not fully capture the complexity of EC reduction in a real-world battery environment, where there are so many interacting components. Yu [51] employed DFT and MD methods to investigate the impact of excess electrons on EC molecules. Their findings revealed that the distribution of electrons becomes localized during dimer formation, resulting in the breakage of C-O bonds and the subsequent production of CO_2_^3−^ ions. The electrolytes used in these experiments are highly complex because of the presence of numerous additives that play a vital role in the formation and growth of SEIs. In another investigation conducted by Wang’s group, both the reduction mechanism of EC and the influence of adding vinylene carbonate (VC) on SEI formation [52] were explored by utilizing theoretical calculations based on DFT. The results indicated that the VC initiated a reduction reaction prior to EC, forming a protective layer to prevent excessive reduction in the electrolyte. Ushirogata [53] utilized the DFT-MD method to investigate the role of VC additives, and the results indicated that VC could replace EC for reduction (Figure 2e). Figure 2e shows that VC has a good substitutability by replacing VC with EC in the decomposition of the anion radical reaction route and results. The DFT-MD method can simulate the reduction reaction process of the VC additive in lithium-ion batteries from the atomic and electronic levels and accurately reveal the specific mechanism of its replacement of EC for reduction, which is helpful to deeply understand how VC forms an SEI film on the negative surface of the battery. At the same time, the method can simulate the behavior of VC under different voltage and temperature conditions, predict its stability and reactivity in the actual battery environment, and provide a theoretical basis for optimizing the battery performance. However, the selection of parameters such as base group and functional in DFT calculation has a significant impact on the accuracy of the results, and the force field parameters in MD simulation also need to be carefully handled, and unreasonable parameter selection may lead to wrong conclusions, which is also the shortcoming of the DFT-MD method. Kevin et al. [24] also studied the modification of SEI through fluoroethylene carbonate (FEC) and found that FEC triggers a reaction leading to the formation of LiF, which enhances the structural stability of SEI. Cresce [54] employed an atomic force microscope (AFM) to investigate the in situ formation process of SEI in the graphite negative electrode (Figure 2f). The force–distance curve (Figure 2f) of the SEI can determine the thickness and mechanical properties of the SEI. Illustrations provide an enlarged view for more accurate measurement of thickness. The dotted line represents the behavior of the probe on the surface without SEI before the formation of SEI as a comparison. AFM can be measured in situ under the actual working conditions of the battery to monitor the dynamic changes in the SEI in real time, which helps to understand the behavior of the SEI under different electrochemical conditions. However, AFM has high requirements for the flatness and cleanliness of the sample surface, and the roughness of the graphite negative surface or pollutants may affect the imaging quality and accuracy of AFM. The SEI exhibits a distinct layered structure, consisting of a soft polymer-like outer layer and a hard inorganic salt inner layer. This structural composition was also confirmed by ex situ XPS analysis. Zhang [55] applied focused ion beam (FIB) to detect the growth and development of the SEI. Initially, the thickness of SEI was measured to be approximately 450–980 nm, displaying a highly uneven surface with non-uniform composition. After multiple charge–discharge cycles, the surface gradually became smoother. However, numerous cracks emerged with a significant increase in the organic matter content, resulting in an overall increase in the SEI thickness to approximately 1200 nm. To further investigate the structural characteristics of SEI, numerous researchers have examined its surface by using AFM [56,57,58], which can distinguish between the SEI and negative substrate based on their distinct mechanical properties. The structure of SEI can be categorized into two layers: a loose, porous organic outer layer and a dense inorganic inner layer [59,60,61,62]. Lu [63] employed an isotope labeling method to analyze the structure of SEI. They initially formed an SEI by using an electrolyte containing ^7^LiClO_4_ and subsequently immersed it in an electrolyte containing ^6^LiBF_4_ (Figure 2g,h). In Figure 2g, the depth distribution of the isotope ratios of ^6^Li^+^ and ^7^Li^+^ in the SEI formed by ^7^LiClO_4_ in the ^6^LiBF_4_ electrolyte at different immersion times (30 s, 3 min, and 15 min) was studied by secondary ion mass spectrometry (SIMS). In the distribution map (Figure 2h), the area with a higher ion signal is shown as a brighter area in the map, which helps to visualize the distribution of different ions in the SEI. In the map, the content and distribution of each ion at different depths can be clearly seen. This is helpful to understand the formation process of SEI and the transport behavior of lithium ions. The isotope labeling method has the advantages of accurate structural analysis and dynamic change monitoring. However, in the actual battery operating environment, complex electrochemical reactions and multi-component interactions may cause the behavior of labeled isotopes to deviate from the ideal situation, affecting the accuracy and interpretability of the results. Their findings revealed that the diffusion process allows the electrolyte to easily penetrate below 5 nm of the SEI surface while only allowing ^6^Li^+^ to pass through the inner layer. This experimental evidence further confirmed the double-layered nature of the SEI.

**Figure 3:**
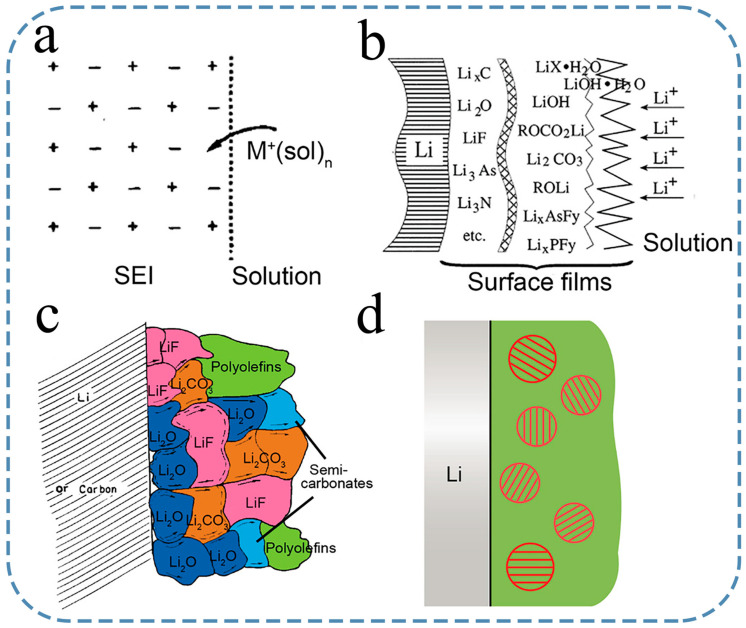
Models of SEI. (**a**) The single crystal representation, which has been reproduced under authorized consent from the copyright holder, The Electrochemical Society, in 1979. (**b**) The multilayer depiction, also reproduced under authorized consent from the copyright holder, Elsevier, in 2000. (**c**) The mosaic concept, reproduced under authorized consent from the copyright holder, The Electrochemical Society, in 1997. (**d**) The plum pudding visualization [68].

The above describes the researchers’ exploration process of SEI components. By using these advanced methods and detection equipment, researchers have initially modeled the composition and structure of SEIs. The first SEI model was proposed by Peled [21] and is shown in Figure 3a. This particular model assumes that the SEI is a single crystal similar to a lithium-ion conductor. And then Peled proposed in 1983 that SEI has a double-layer structure model, with a dense inner layer near the Li metal side and a porous outer layer near the electrolyte side. However, the subsequent experimental investigations have demonstrated that the structure of SEI is much more intricate and dynamic than originally assumed. By using Raman spectroscopy, FTIR, and XPS techniques, Aurbach [59] made a significant discovery on the chemical composition of SEI. Their findings revealed that the SEI was composed of a combination of diverse organic and inorganic species. Consequently, they proposed the new double-layer model, suggesting that the SEI consists of an inner layer rich in inorganic components (in contact with the lithium metal) and an outer layer abundant in organic substances (in contact with the electrolyte), as illustrated in Figure 3b. It is speculated that the inorganic compounds, such as LiF and Li_2_O, exhibit greater chemical stability against lithium than the organic compounds. As a result, they tended to accumulate on the lithium surface. For example, Zhou et al. [64] utilized operando liquid secondary ion mass spectrometry to detect that the SEI formed in a Li bis(fluorosulfonyl)imide (LiFSI)-DME electrolyte consists of a thin, compact, and continuous layer rich in Li_2_O, along with an outer layer composed of loosely connected oligomers/polymers.

Subsequently, the mosaic model was proposed as an improvement on the layered model [33]. The mosaic model builds upon the concept that the SEI consists of both dense inner and porous outer layers, indicating that each component forms a distinct microphase, which together creates a mosaic-like structure within the SEI and is similar to Aurbach’s research. A visual representation of this concept is shown in Figure 3c. The mosaic model became widely accepted as the leading theory for explaining the structure of SEIs, mainly because it aligns well with the XPS results, ionic conductivity measurements, and the logical ranking of the chemical stability of different SEI species in relation to lithium [63]. However, recent observations from the cryogenic transmission electron microscopy (cryo-TEM) have posed challenges to the generally accepted layered mosaic model [65]. According to literature report [66], it has been confirmed that the SEI primarily consists of an amorphous phase, regardless of the type of electrolyte used in the batteries. Furthermore, there is no significant accumulation of crystalline microphases on the surface of lithium metal. In certain instances, these crystalline microphases are randomly dispersed within the SEI, while in other cases, they tend to concentrate predominantly on the outer layer [67]. The cryo-TEM observation reveals that the SEI structure bears resemblance to the famous “plum pudding model” (Figure 3d), with the amorphous phase resembling the pudding and the embedded crystalline microphases resembling plums [68].

The development of structure models undoubtedly provides an important contribution to the further study of SEIs. These models will also be gradually improved with the development of science and technology and will provide important theoretical guidance for the design and construction of SEIs for lithium metal batteries with better performance.

### 3.2. Development of SEI Growth Prediction Model

**Figure 4:**
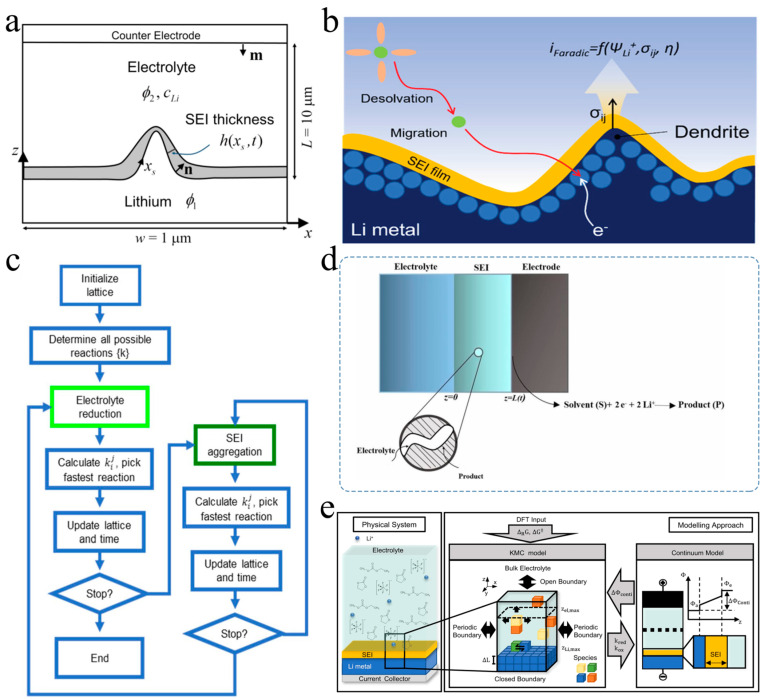
Models and schematic diagrams of the formation process of SEI on the surface of the lithium metal cathode. (**a**) Schematic representation of the model. The lithium metal substrate at the bottom serves as the negative electrode, characterized by a dynamic boundary influenced by the rate of lithium deposition. The profile of the substrate surface is defined by the function z = z(x, t). Additionally, there exists an evolving SEI on top of this surface with a varying thickness (coverage density) denoted as h (xs, t) [69]. (**b**) Schematic of electrodeposition of Li at the interphase between the bulk metal electrode and SEI involving electrochemical kinetics of Li as well as mechanical stresses [2]. (**c**) Flow diagram under which the algorithm operates. The light-green loop addresses the electrolyte reduction reactions, while the dark-green loop addresses the exchange reactions driving the SEI aggregation processes [77]. (**d**) Schematic representation of the SEI formation process occurring at the interphase between the SEI and electrode. The SEI allows for continuous diffusion of solvent (S) and lithium ions from the solution phase, which subsequently react with electrons present on the surface of the electrode. This reaction ultimately leads to the generation of an insoluble product (P) [78]. (**e**) Reconceptualizing the formation of SEI on lithium metal through schematic modeling [79].

In recent years, several emerging mathematical models have been developed for predicting the growth and formation of the SEI. Liu [69] established a model combining the formation of lithium dendrites with SEI (Figure 4a). The growth of dendrites affects the curvature and stretching of the SEI film layer, thereby influencing its evolution. Meanwhile, the growth of SEI alters the resistance in the battery and the reactive current density in the circuit, thus affecting the formation and growth of lithium dendrites. The effects of current density, SEI resistivity, defects, and inhomogeneity of the lithium metal surface on the formation and growth of lithium dendrites were studied by using this mathematical model. In addition, their research can be extended to explore various other phenomena associated with the simultaneous growth of SEI and dendrites. These studies provide insight into the underlying mechanisms of dendrite growth and facilitate the improvement of SEI design strategies.

Liu [2] presented an electro-chemo-mechanical model (Figure 4b) that combines the electrochemical kinetics and mechanical behavior to investigate the design of artificial SEIs for uniform lithium electrodeposition. The model is based on a modified Butler–Volmer equation, incorporating the coupling potential, mechanical stress, and Li-ion concentration fields to describe the interfacial dynamics between the SEI and the lithium metal anode. The model is implemented by using a phase-field approach and applied to a structured substrate covered with an SEI. This framework allows for an independent evaluation of the SEI’s ionic conductivity and Young’s modulus, highlighting their crucial roles in governing the morphology and uniformity of Li electrodeposition. The predictions based on this model are validated by the experimental data in the literature [70,71,72,73,74], providing a comprehensive understanding for optimizing artificial SEIs in LMBs. Additionally, the model and computational methodology developed in this work are broadly applicable and can be extended to the design of protective layers for other metal anodes [75], contributing to the advancement of next-generation energy storage systems.

Li et al. [76] elucidates the interphase evolution mechanism concerning the impact of thermal distribution on the SEI using a thermal distribution evolution model. Initially, the effect of SEI’s diffusivity on its thermal distribution was investigated by varying the SEI diffusion coefficient. The results demonstrate that when the ratio of SEI diffusion coefficient to electrolyte diffusion coefficient is 5, the growth of lithium dendrites is minimized, and the temperature gradient at the dendritic tip remains relatively low. This coefficient is influenced by the electrolyte composition, especially by the ions, as discussed below. Subsequently, the influence of cation and anion diffusion coefficients on the thermal distribution within the SEI is explored by changing the diffusion rates of ions through the SEI. It is found that when the SEI’s diffusivity relative to the electrolyte is low, the anions play a significant role in modulating the heat distribution within the SEI, exhibiting a threshold effect. Specifically, an increase in the anion diffusion coefficient within a certain range leads to a reduction in the temperature gradient, while surpassing this range results in an elevated temperature distribution. Finally, the introduction of anisotropy in the SEI structure facilitates the lateral growth of lithium dendrites and reduces the temperature gradient at the dendrite tip, promoting a more uniform thermal distribution in the SEI. These findings provide critical insights into the design of SEI and offer guidance for enhancing the performance and stability of LMBs.

Perez-Beltran [77] presented a detailed mechanistic model for the formation of the SEI and the electrochemical plating and stripping of lithium ions on a lithium metal anode during cycling by using a first-principles kinetic Monte Carlo (kMC) approach (Figure 4c). The LiFSI-F5DEE electrolyte system, which consists of a 1.2 M lithium bis(fluorosulfonyl)imide (LiFSI) salt dissolved in a fluorinated ether solvent, 1-(2,2-difluoroethoxy)-2-(2,2,2-trifluoroethoxy)ethane (F5DEE), was used as the research object. The findings reveal that the SEI’s composition and structure are strongly influenced by the aggregation of organic and inorganic components, which compete for dominance. The grain boundaries between different inorganic SEI phases, such as LiF and Li_2_O, are identified to be faster pathways for lithium-ion transport, enhancing the overall conductivity of the SEI. Additionally, the efficiency of lithium reduction during plating decreases over time due to the densification of the SEI and stronger ionic interactions within the inorganic bulk phase. The simulated SEI evolution well agrees with the experimental observations, providing a predictive framework for future studies on SEI formation and electrolyte design. Their work underscores the importance of dynamic SEI modeling in advancing high-energy-density LMBs.

A growth model for the SEI was presented by Kamyab [78], which considers both the impact of the solvent reduction reaction kinetics at the SEI/electrode interphase and the solvent diffusion through the SEI (Figure 4d). The numerical solution of the governing equations enables the prediction of the solvent concentration distribution, SEI thickness, and capacity loss. By fitting the capacity loss predictions to the two sets of experimental data from previous studies, it is evident that the mixed-mode model effectively describes the capacity loss in cells resulting from the SEI growth on the anode under constant-voltage conditions.

Wagner-Henke et al. [79] presented an extensive analysis based on the kMC model and the continuum model to investigate the initial SEI formation on the lithium metal surface in a carbonate-based electrolyte (Figure 4e). Their research explored a larger scale in terms of length and time compared with the similar studies conducted by using molecular dynamics simulations. This model, which combines the multiscale kMC and continuum approaches, demonstrates the presence of a SEI primarily composed of LiF on top of Li_2_CO_3_ and Li after 1 µs. The formation of this SEI can be attributed to the intricate interactions between various electrolyte components and the salt decomposition processes. Additionally, they observed that a more fragmented SEI with a certain organic content was formed when the local concentration of Li ions was low. Conversely, increasing the salt concentration leads to a faster passivation process on the lithium metal surface. These findings provide valuable insights for designing SEIs on the lithium metal surface and represent an important advancement towards the knowledge-based engineering of SEIs.

In general, the rational use of mathematical models can provide a better prediction of the various properties of the SEI and optimize the experimental schemes. Therefore, it is of great significance to combine the mathematical modeling with the research of SEI in LMBs, that is, to combine theory with practice to obtain twice the result with half the effort. Although there are some differences between the SEIs of lithium-ion batteries (LIBs) and LMBs, the SEI mathematical models of LIBs and LMBs have many similarities. Firstly, the SEI formation process is critical for both battery systems, involving the electrochemical reactions and material diffusion. For example, Wagner-Henke [79] and Phul [80] both referred to the growth process of SEIs and the effect of potential decline on capacity decay from the perspective of two different types of batteries, respectively, suggesting that the SEI’s mathematical model can be used in both types of batteries to predict and analyze the growth and performance of SEI. In addition, the structure and composition of SEIs are also similar in both LIBs and LMBs. Adenusi [81] and Tan [82] highlighted the multilayer structure and dynamic changes in the SEI in both lithium-ion batteries and lithium metal batteries, which are critical to understanding the mechanism of the SEI model and improving the cyclic stability and safety of batteries. In addition, Tan [83] reported that the multilayer structural model of SEI has been widely adopted in the study of graphite anode surfaces, which is also applicable in the lithium metal anodes. The SEI mathematical models of LIBs and LMBs have many similarities in research, including the formation process, the structural composition, and the establishment method of the mathematical model. These common points provide an important theoretical basis for an in-depth understanding of the performance and optimal design for these two types of batteries.

This section introduces the development of structural and mathematical models of the SEIs and their application scenarios and ranges. There is no doubt that they have made significant contributions to the development of this field [84,85]. The above discussion is based on the liquid electrolyte batteries, but the formation of SEI in liquid electrolytes and solid electrolytes is fundamentally different. In the liquid electrolyte batteries, the SEI is usually formed by the reaction of the electrode material and the electrolyte at the solid–liquid phase interphase and has the characteristics of a solid electrolyte, which is an electronic insulator but an excellent conductor of Li^+^. While in the solid electrolyte batteries, the formation of SEI involves more complex chemical and electrochemical reactions, and its structure and composition may be dynamic and complex [86]. Therefore, relatively little research has been performed on the research of SEI models in the solid-state LMBs. Recently, Li [87] developed a mathematical model that integrates the physical and chemical processes that form dendrites. In addition, the model provides a new insight that exchanging certain properties in a new electrolyte can slow down or even completely inhibit dendrite formation, which makes an important contribution to the SEI modeling of LMBs with solid electrolytes. A better understanding of the growth process of lithium dendrites in solid-state batteries will help us to build a stable SEI to avoid the growth of lithium dendrites. Using MD simulations guided by machine learning potential, Ren et al. [88] studied the formation and growth mechanism of SEI at the atomic level. It identifies four stages of evolution: rapid ion diffusion, nucleation, Li_2_S growth, and stability. These insights into the formation of SEI are critical to improving the durability of all-solid-state batteries (ASBs), especially at the interphase between lithium metal and solid electrolyte. Because the research of Ru et al. is based on the specific solid electrolyte, whether it can be extended to other solid electrolytes remains to be studied. But there is no doubt that the model helps to optimize battery performance and life by understanding the kinetic and thermodynamic processes that control the development of SEI. There are also other studies [88,89] of SEI films in all-solid-state batteries; however, the solid electrolyte protective layer still needs a lot of research and exploration.

However, the degree of alienation of each model is increasing, and the background is very different, so the realization of practical applications will be relatively distant. Therefore, determining whether the model can be applied to actual production is a huge challenge. The emerging artificial intelligence (AI) technology has certain advantages in the mathematical models [89,90,91,92,93], which can improve accuracy and simplicity. At present, there are also some studies [94,95] in this field, which is the trend of future development. The research and development of LMBs and their materials is essentially a process of exploring scientific problems based on the basic physics theories. The application of theoretical simulations to explore the electrochemical reaction mechanism of the energy and matter conversion in the process of energy storage can effectively improve the design of SEIs for LMBs. Through physical basis and theoretical simulation, the structure-activity relationship can be optimized, the practical problems in production and use can be solved, and thus a reliable and valuable theoretical basis can be provided. Regardless of the mathematical model, its root is to solve the interaction potential function between the atoms or units. Owing to the evolution of computational methods at different physical scales, the gradually emerging empirical and modified parameters in computational physics models affect the accuracy of the simulation results and the efficiency of the calculation process. With the increase in simulation data [96] and attention to computational efficiency, the introduction of machine learning has realized the need to balance high efficiency and throughput. Many excellent reports [97,98,99,100,101] on machine learning and AI have emerged, making a significant contribution to solving the problem of LMBs. Research methods have gradually changed from data-driven to intelligence-driven, and the research paradigm of new energy has gradually evolved from qualitative analysis to quantitative prediction [102]. From independent research at different scales to multiscale coupling research, cross-scale correlation research has been conducted to explore the common principle of electrochemical energy storage.

## 4. Construction of Functional SEIs

The primary problem hindering the practical application of LMAs is the growth of lithium dendrites. The most straightforward solution is to construct an effectively stable SEI. An ideal SEI should possess exceptional structural uniformity and exhibit favorable lithium-ion conductivity, surface electronic insulation, and mechanical strength. This layer plays a crucial role in enhancing the homogeneous nucleation of lithium, facilitating the efficient transportation of lithium ions, preventing the local aggregation of lithium, and thus promoting the uniform deposition of lithium. Additionally, it is essential for the SEI to remain intact during cycling to inhibit dendrite growth and impede the passage of solvent molecules, thereby slowing down undesirable side reactions involving lithium metal. Researchers have employed various strategies, such as constructing functional inorganic, organic, and organic/inorganic composite SEIs and other modifications [103] on the surface of lithium metal to improve the performance of LMAs.

### 4.1. Artificial Inorganic SEI

As a pioneer in the field of SEI composition studies, Zhang et al. [104] discovered that SEIs were composed of intricate mixtures for the first time. Through the utilization of FTIR and XPS, they found that the SEIs formed in electrolytes with lithium hexafluorophosphate (LiPF_6_) dissolved in organic carbonate solvents were composed of various species, including lithium semicarbonates (ROCO_2_Li), lithium dicarbonates ((ROCO_2_Li)_2_), LiF, Li_2_CO_3_, Li_2_O, and a small quantity of lithium fluorophosphate (LiPO_x_F_y_) [105]. And a large number of subsequent studies have also discovered that LiF, Li_2_O, and Li_2_CO_3_ are indispensable components for the electrochemical cycling of lithium batteries in PC or EC electrolytes [106]. The following is an overview of the latest research on inorganic SEIs.

**Figure 5:**
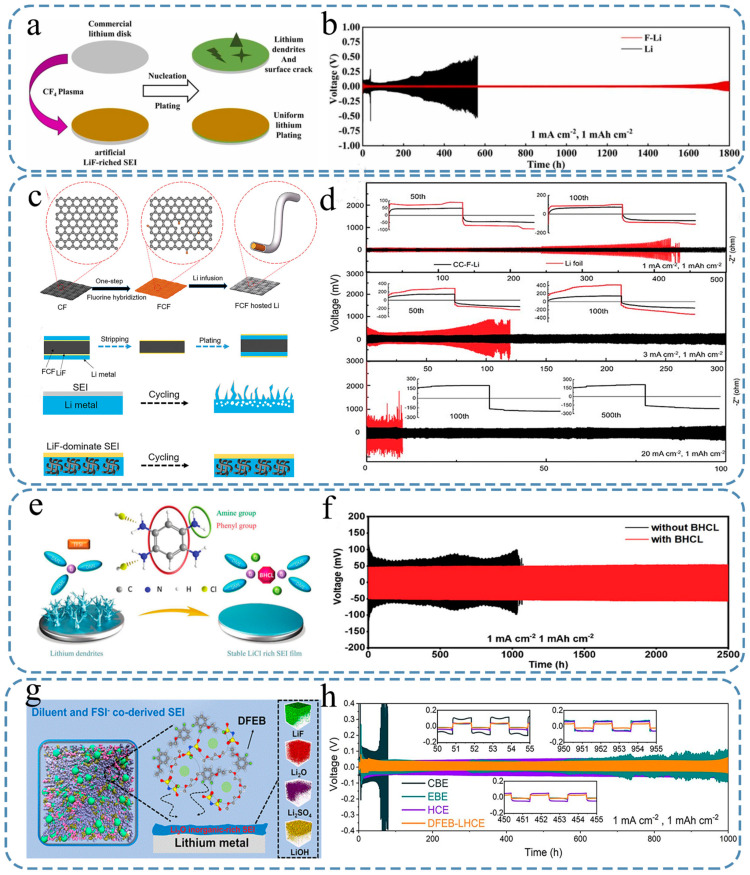
Effects of different organic components on lithium metal batteries and SEI synthesis diagram. (**a**) Comparison chart with and without CF_4_. (**b**) The performance of symmetrical cells with F-Li||F-Li and Li||Li configurations during cycling [107]. (**c**) The process flow chart of the preparation of FCF carrier lithium and the deposition mechanism of FCF on different metal lithium. (**d**) Galvanostatic cycling performance of lithium symmetric cells at different current densities [109]. (**e**) Molecular structure of BHCL and its designing principle. (**f**) Galvanostatic cycling performance of lithium symmetric cells in the presence and absence of BHCL [111]. (**g**) SEI main components schematic. (**h**) The cycling stability performance of Li||Li half-cells was evaluated under various electrolytes at a current density of 1 mA cm^−2^ and a capacity of 1 mAh cm^−2^ (inset: magnified curves for reference) [113].

Zhou et al. [107] employed a CF_4_ plasma technology to introduce an artificial fluoride-rich SEI on the surface of the lithium metal anode (Figure 5a). The stability of the electrode can be enhanced by the high mechanical strength of the LiF-rich SEI while reducing the ratio of cracks. In addition, uniform nucleation was guided by lower diffusion, achieving the purpose of inhibiting lithium dendrites. The Li||Li symmetric battery consisting of F-Li exhibited stable cycling performance for 1700 h (at a current density of 1 mA cm^−2^ and a capacity of 1 mAh cm^−2^) and 1400 h (at a current density of 2 mA cm^−2^ and a capacity of 2 mAh cm^−2^), with minimal overpotentials observed at approximately 13 mV and 80 mV, respectively (Figure 5b). Coincidentally, Han et al. [108] also conducted in-depth research on the ability of LiF to prevent electron tunneling, further confirming the significance of the LIF-rich interphase protective layer.

In the study conducted by Xia et al. [109], they successfully achieved both volumetric accommodation and stable SEI by utilizing a metal-free fluorinated carbon fibers (FCF) host, which was prepared via a simple one-step fluorination method. By using the molten lithium infusion technique, they fabricated a highly stable SEI dominated by LiF on the FCF host. Unlike previous approaches involving fluorinated electrolytes, additives, fluorinated salts, or pre-reactions of lithium foil with polymeric fluorine donors, the thin and uniform LiF-dominant SEI formed mainly from the FCF host plays a crucial role in regulating homogeneous lithium nucleation and enabling efficient charge transfer at the electrode/electrolyte interphase. Figure 5c illustrates the preparation process of FCF-hosted lithium and the mechanisms of lithium deposition on the different types of LMAs. It also elucidates the formation mechanism by which LiF is predominantly present in the SEI, provides insights into how lithium is deposited onto the LMAs with the SEIs with or without LiF, and shows structural and morphological changes during this process. The LMA hosted by FCF exhibited improved coulombic efficiency (Figure 5d), uniform lithium deposition, and excellent stability during high-rate cycling for over 100 h at a current density of 20 mA cm^−2^ and 1 mAh cm^−2^. Additionally, when combined with cathodes such as LiNi_0.8_Co_0.1_Mn_0.1_O_2_, sulfur, or thick LiCoO_2_, the full cells demonstrated enhanced rate capabilities and prolonged cycling durability, even under low electrolyte conditions. This study confirms the practical applicability of utilizing FCF to host lithium and presents an innovative approach for cost-effective and high-performance lithium metal batteries.

Yang et al. [110] employed a straightforward method involving InCl_3_ to polymerize 1,3-dioxolane (DOL) and construct a stable LiF/LiCl/LiIn hybrid SEI. This was confirmed by analyzing the XPS data and observing the structure using cryo-TEM. Additionally, DFT calculations and finite element simulations (FES) were conducted to validate the excellent insulating properties of electrons as well as the efficient transport capabilities of lithium ions exhibited by this hybrid SEI. Furthermore, it was observed that the interfacial electric field demonstrated an evenly distributed potential and facilitated a higher flux of lithium ions, resulting in the uniform deposition of lithium without dendrite formation. The application of this LiF/LiCl/LiIn hybrid SEI in the Li||Li symmetric batteries showcased consistent cycling performance over 2000 h without any instances of short circuits at a current density of 1 mA cm^−2^. Moreover, this hybrid SEI also displayed exceptional rate capability and remarkable stability during cycling tests with LiFePO_4_/Li batteries, achieving a high specific capacity of 123.5 mAh g^−1^ at a 10 C rate.

Lin et al. [111] reported for the first time the utilization of a tetrahydrochloride form of 1,2,4,5-benzenetetramine with high amine content (BHCL) to effectively control the solvent sheath and construct a LiCl-rich inorganic SEI (Figure 5e). BHCL possesses four amine groups with a high donor number, enabling its strong coordination with lithium ions and involvement in the electric double layer near the lithium metal. Subsequently, the chloride ions attached to the amine group penetrate the solvent sheath and decompose to generate an inorganic SEI abundant in LiCl. The Li||Li symmetric battery with modifications of this SEI demonstrated stable cycling for more than 2500 h at a current density of 1 mA cm^−2^, while maintaining an overpotential of approximately 45 mV (Figure 5f). The performance of both the Li||Cu and Li||LFP cells also showed significant improvement.

Beichel et al. [112] successfully utilized fluorinated organic Brønsted acid HBFEP to modify the surface of lithium metal, enhancing its cycling performance in rechargeable batteries. By immersing the lithium anode into a diluted solution of phosphoric acid derivative (CF_3_CH_2_O)_2_P(O)OH (HBFEP) with a concentration of 0.05 to 0.20 M, an artificial SEI was formed on the surface of lithium metal. Subsequently, HBFEP reacts with the lithium surface, resulting in the formation of lithium salt (LiBFEP), which acts as an inorganic coordination polymer capable of conducting lithium ions. In addition, this film possesses self-healing properties owing to its ability to break and reform ionic bonds when the lithium volume expands during cycling. The Li||Li symmetric battery with HBFEP-modified lithium electrodes exhibits a significant increase in cycle lifetime, ranging from three to nearly four times longer at 0.1 mA cm^−2^. This suggests that the LiBFEP-enriched layer acts as a protective barrier, facilitating lithium-ion conduction between the lithium electrode and the electrolyte, thereby enhancing the rechargeability of the lithium electrodes.

Although the current research direction is mainly LiF (the main components of SEI are shown in Figure 5g), Zeng et al. [113] designed a super-coordinated co-solvated diluent, 2, 3-difluoroethoxy-benzene (DFEB), to control the solvation structure so that the diluent and anion participate in the formation and growth of SEI. It is customized for the lithium metal anode with Li_2_O, which is rich in inorganic SEI. They also found that high-concentration electrolytes (HCEs, typically lithium salt concentrations above 3 M) and localized high-concentration electrolytes (LHCEs) can effectively improve the long-term cycling performance of LMBs. Then, they assembled the DFEB into a lithium battery in the environment of LHCEs (DFEB-LHCE), in which DFEB participates in the first solvated shell and collaborates with FSI^−^ to form a Li_2_O-dominated inorganic-rich SEI, while the LIF-dominated SEI was formed in LHCE where the traditional diluents do not participate in the solvated structure. Owing to this unique SEI structure, a high coulombic efficiency (CE) of 99.58%, low nucleation overpotential, and dense uniform lithium deposition were achieved in the Li||Cu half-cells. As a result, the formation of lithium dendrites in the lithium symmetric batteries can be effectively inhibited by the non-in situ and in situ lithium deposition morphology observations. More importantly, in full cells with LFP, NCM811, and sulfurized polyacrylonitrile (SPAN) as cathodes, DFEB-LHCE can achieve a more stable long-cycle performance, and the Li||LFP full battery using DFEB-LHCE maintained 85% of its capacity after 650 cycles with a stable CE of 99.9%. Moreover, the 1.5 Ah lithium metal soft-pack battery using DFEB-LHCE still exhibited an excellent capacity retention rate of 89% and an average CE of 99.93% after 250 cycles. This work reveals the superiority of the Li_2_O-dominated SEI in stabilizing lithium metal anodes and provides a new perspective for customizing the composition of the SEI by regulating the solvation structure through co-solvation diluents. Furthermore, the DFEB-LHCE exhibited exceptional lithium compatibility and interfacial stability, as evidenced by its minimal polarization voltage of approximately 65 mV and consistent cycling performance for a duration of 1000 h with negligible fluctuations (Figure 5h).

In general, the conductivity and stability of lithium batteries can be significantly enhanced by the presence of an inorganic SEI. The inorganic SEI can selectively conduct lithium ions, provide fast lithium ion transport channels, and avoid polarization during ion transport, thereby improving the conductivity of the battery [114]. At the same time, it has high chemical stability and low reactivity with the electrolyte, which can reduce harmful side reactions and maintain the long-term stability of the battery [115]. Swastik [106] has reported unconventional advancements in the transport capability of lithium ions and mechanical stability within amorphous structures of crucial sub-SEI components, such as LiF, Li_2_O, and Li_2_CO_3_, using first-principles calculations. The density functional theory based on first principles was employed by Xu et al. [116] to investigate the electrochemical characteristics of artificial SEIs composed of lithium halides (LiF, LiCl, LiBr, LiI). The surfaces were examined for the adsorption and migration behavior of lithium ions while analyzing and comparing the structural stability, mechanical properties, and electronic insulation properties of the SEIs. Furthermore, a study was conducted on the adsorption and migration behavior of lithium ions on these surfaces. Ultimately, it was observed that among these films, LiF exhibited exceptional stability in both body and surface structures. It also demonstrates a stable interphase structure with lithium metal, along with superior mechanical properties, electronic insulation capabilities, and inhibition potential against lithium dendrite growth. Consequently, it can be concluded that LiF serves as an ideal artificial SEI for applications involving lithium metal. Many current studies [117] on artificial SEIs contain LiF components, which shows the importance of LiF. However, Xu et al. [118] developed an interphase of the SEI dominated by inorganic CsFSI without LiF, further proving that LiF is not necessary for an artificial SEI. Hobold [119] also compared two SEIs containing Li_2_O and LiF under certain conditions and found that the SEI containing Li_2_O showed better battery performance. This shows that the study of the inorganic components of the artificial SEI should not be too limited to LiF, but the important role of LiF should not be ignored.

### 4.2. Artificial Organic SEI

A well-performing SEI is essential for reducing battery resistance, minimizing polarization, and ensuring stable charge and discharge. Two main factors affect the performance of the organic SEI: the thickness of the SEI film and the effect of the doping substances. Firstly, the utilization of a thin and lightweight SEI aids in mitigating the power degradation, enhancing the initial capacity, and prolonging the battery lifespan. Secondly, the doping substances have the potential to affect the composition and structure of organic compounds or their presence within the SEI and thus influence the characteristics of LMAs [120].

**Figure 6:**
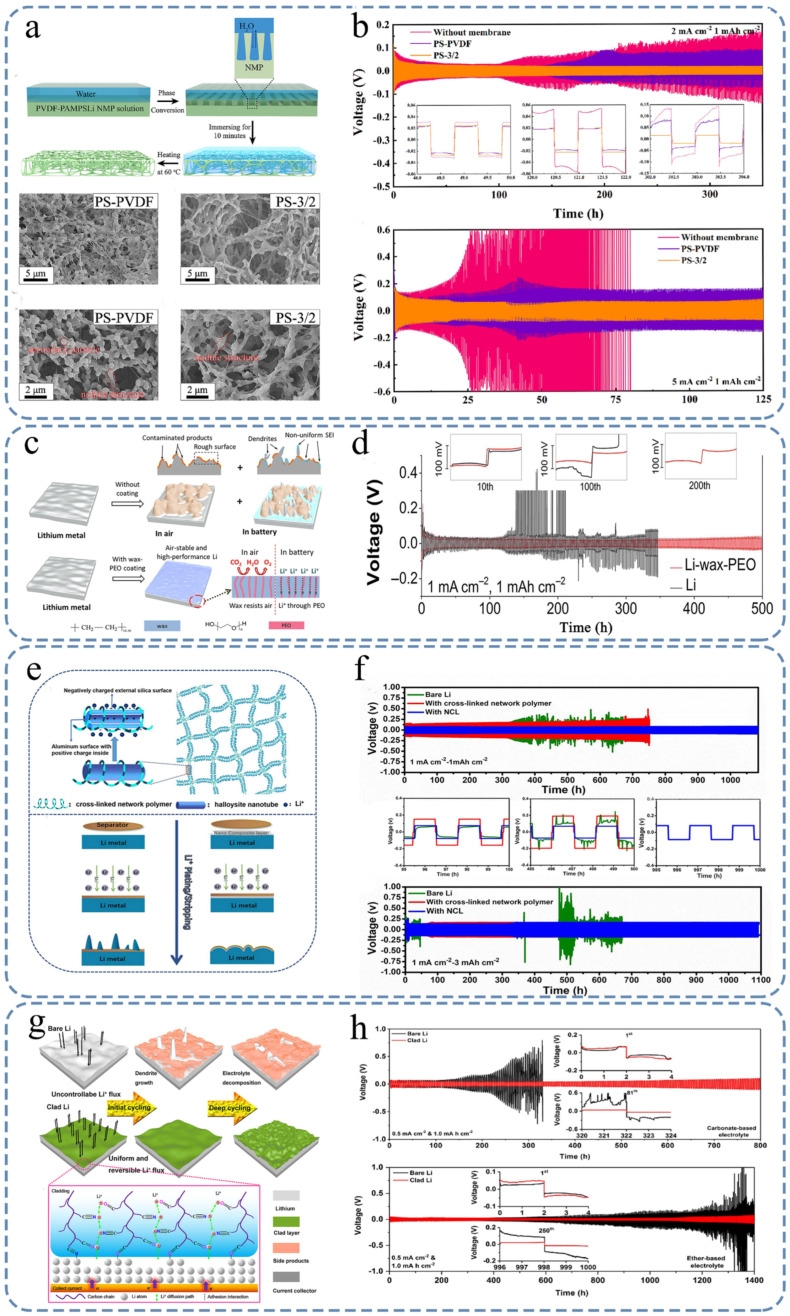
Effects of different inorganic components on lithium metal batteries and SEI synthesis diagram. (**a**) Schematic representation of the experimental procedure employed for PVDF-PAMPSLi and the SEM utilized to capture images of the PS-PVDF and PS-3/2 membranes. (**b**) Evaluation of cycling efficiency in Li/PS-3/2/Li, Li/PS-PVDF/Li, and Li/Li batteries under varying current density conditions [121]. (**c**) Representation illustrating the operational aspects of the wax-PEO coating. (**d**) Galvanostatic cycling performance of the Li symmetric batteries [123]. (**e**) Microstructure diagram of NCL. (**f**) Schematic diagram of the mechanism evolution of NCL-Li and bare Li electrodes during the plating/stripping process [124]. (**g**) Schematic depictions depicting the process of repetitive plating and stripping. (**h**) Galvanostatic cycling performance of the Li symmetric batteries [125].

Wu [121] used a phase separation method (as shown in Figure 6a) to fabricate a porous PVDF-lithium poly (acrylamide-2-methyl-1-propane-sulfonate) (PAMPSLi) composite membrane. Figure 6a shows the detailed process of preparing the SEI and the SEM utilized to capture images of the PS-PVDF and PS-3/2 membranes. In this SEI, PVDF provides a three-dimensional porous framework, and PAMPSLi provides lithium-ion conduction. Through the modification of this composite film, the Li-ion transfer number was significantly increased, and the LMBs possessed a fast Li^+^ transport pathway, thus improving the stability of the LMA and slowing the consumption of liquid electrolytes. The PVDF-PAMPSLi-protected symmetric cells exhibited a consistent cycling performance for over 350 h at a current density of 2 mA cm^−2^ and a charge/discharge capacity of 1 mAh cm^−2^. When operated at a higher current density of 5 mA cm^−2^ (Figure 6b), the cell exhibited stable cycling behavior for 125 h with an overpotential maintained at ~50 mV. In the Li||Cu half-battery tests, the Cu electrode modified with PVDF-PAMPSLi showed an excellent CE of 97.43% over 265 cycles at 2 mA cm^−2^ (Figure 6b). Furthermore, when PVDF-PAMPSLi-coated lithium was used in LFP||Li and NCM811||Li full cells under low electrolyte conditions (10 μL mAh^−1^), significantly enhanced cycling stability and improved discharge capacities were achieved.

Kang et al. [122] formed SEI by coating the surface of lithium with an organic layer through a spontaneous reaction between lithium and carboxylic acid. This newly formed carboxylate layer effectively prevented excessive lithium deposition and inhibited dendrite growth. The uniformity of the organic layer plays a crucial role in achieving stable cycling performance. By maintaining a consistent 1 μm thickness of the carboxylate layer, the lithium electrode demonstrated excellent durability during charge–discharge cycling at 0.5 mA cm^2^ for up to 1000 h. DFT calculations revealed that the oxygen atoms in lithium carboxylate contribute to enhanced lithiophilicity and reduced deposition overpotential. This study presents a practical approach to utilizing LMAs in various applications.

To overcome the challenges faced in practical applications of LMA, such as dendrite formation and adverse reactions due to high activity when exposed to air or electrolyte, Zhang et al. [123] have developed a wax-based coating technique. Using ion-conducting poly (ethylene oxide) and dip-coating technology, they were able to create an air-stable and waterproof SEI surface on the LMA (Figure 6c). Figure 6c shows in detail the detailed process of preparing the SEI and the schematic diagram of the comparison with the bare Li. This allowed for stable performance even after 24 h in ambient air with 70% relative humidity while retaining approximately 85% of its electrochemical capacity. Additionally, the coated LMA exhibited no significant adverse reactions or capacity decay when exposed to water. Their composite coating also enabled steady cycling performance for up to 500 h in the Li symmetric cells (Figure 6d) and a low capacity decay rate of only 0.075% per cycle after 300 cycles in lithium–sulfur batteries assembled with the packaged anode.

Liu et al. [124] developed a unique nanocomposite layer called NCL, which combines lithiated halloysite nanotubes (HNT) and a polyethylene oxide (PEO)-based cross-linked network polymer, as shown in Figure 6e. And Figure 6e illustrates the deposition/stripping processes of lithium on the NCL-Li and bare lithium electrodes. As the protective layer of LMA, the NCL inhibits the side reactions between the lithium metal and liquid electrolyte, promotes the diffusion kinetics of lithium ions, and regulates their uniform deposition to achieve dendrite-free morphology. The symmetrical cells with the lithium electrode protected by NCL exhibited a stable cycling performance for over 1000 h and 1100 h at 1 mA cm^−2^ under cycling capacities of 1 mAh cm^−2^ and 3 mAh cm^−2^, respectively (Figure 6f). In addition, the half-cells consisting of the NCL-Li electrode demonstrated dendrite-free and reversible Li deposition with an impressive coulombic efficiency of 99% for up to 170 cycles at 0.5 mA cm^−2^. Moreover, the LiFePO_4_ full cell successfully achieved a good capacity of 115 mAh g^−1^ with a sensational capacity retention of 97.5% over 800 cycles at 2C.

In the study conducted by Wang et al. [125], they propose a novel approach to address this issue (Figure 6g). Figure 6g shows in detail the schematic diagram of the LMA covering the SEI and the bare Li plating and stripping process. Their method involves the simultaneous control of the flow of lithium ions and regulation of the activity on the surface of lithium metal through the utilization of a terpolymer SEI. This layer effectively guides the movement of lithium ions while minimizing undesired reactions during the lithium plating/stripping processes, resulting in a smooth surface for the LMA and enhanced electrochemical performance. Notably, impressive cycling durations of 800 h in carbonate-based electrolytes and 1400 h in ether-based electrolytes were achieved for the lithium symmetric batteries (Figure 6h). Additionally, the asymmetric Li-LiFePO_4_ and Li-sulfur cells demonstrated prolonged cycle lifespans with reduced interfacial resistance after repeated cycling. This method significantly improved the electrochemical performance of lithium metal batteries by effectively guiding the movement of lithium ions and reducing adverse reactions. Its excellent performance in different electrolyte systems indicates that this method has broad application prospects in the field of LMBs.

The development of an inorganic SEI adheres to a growth model that combines both two-dimensional and three-dimensional mechanisms, and its formation is greatly influenced by the overpotential. A higher overpotential promoted the formation of a two-dimensional SEI. On the other hand, organic SEI strictly follows a two-dimensional instantaneous nucleation and growth model, resulting in epitaxial passivation of electrodes [126]. This unique characteristic of the organic SEI provides elasticity to alleviate significant volume changes during repeated cycling, effectively preventing the formation of lithium dendrites and improving the stability of LMBs [127]. Additionally, by utilizing the strong binding ability and exceptional stability of the Li polyacrylic acid (LiPAA) polymer, the improved SEI can effectively reduce the occurrence of side reactions, thereby inhibiting the growth of lithium dendrites and solving safety issues [128]. A plausible approach to achieve high energy density, enhanced safety, and prolonged cycle life in LMB is through the rational design of polymer-based artificial SEIs [129]. However, further investigations are imperative to advance our understanding in this domain.

### 4.3. Artificial Inorganic/Organic Composite SEI

For the above description, we can understand that both the organic and inorganic SEIs can effectively protect the LMAs, so whether a composite layer composed of organic and inorganic species can play a better role? The answer is yes. They [130,131,132,133,134,135,136] have been extensively studied for their high mechanical strength, high lithium-ion conductivity, and electron-insulating properties.

**Figure 7:**
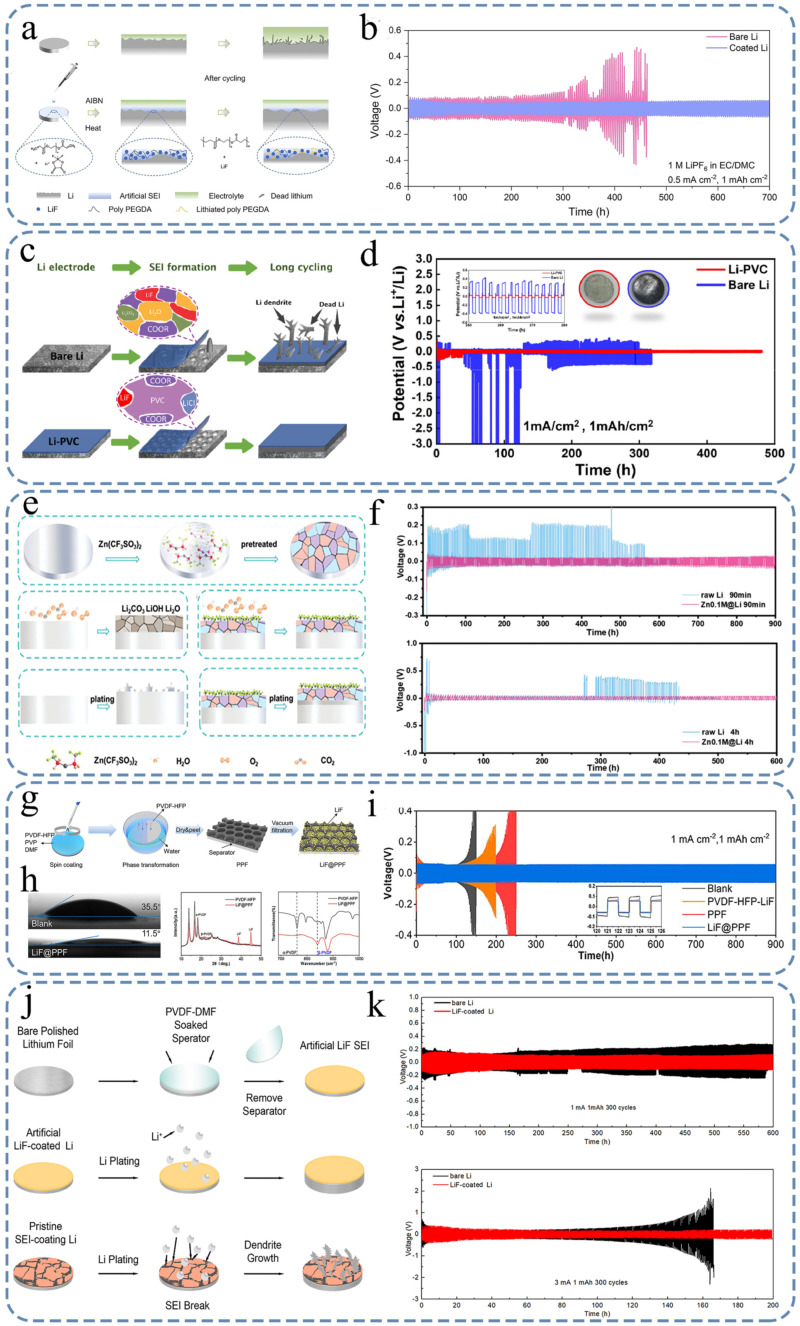
Effects of different organic/inorganic components on lithium metal batteries and SEI synthesis diagram. (**a**) Schematic illustration of organic-inorganic composite SEI preparation through in situ polymerization and the corresponding Li deposition behaviors of bare Li and modified Li electrodes. (**b**) Electrochemical behavior of Li||Li symmetric cells under a current density of 0.5 mA cm^−2^ and a charge capacity of 1 mA h cm^−2^ is investigated using both artificially protected Li with an artificial SEI and bare Li [130]. (**c**) Image depicting the morphological evolution in lithium and lithium-PVC composites. (**d**) After exposure to air, the voltage profiles of a Li||Li symmetric battery with symmetrical electrodes were recorded under a current density of 1 mA/cm^2^, while maintaining a capacity of 1 mAh/cm^2^ [133]. (**e**) Schematic diagram of the preparation process of a lithium sheet containing Zn(CF_3_SO_3_)_2_ and an experimental diagram of a comparison with naked Li. (**f**) Reversible cell performance of pristine the Li||Li symmetric batteries coated with 0.1 m Zn (Zn0.1m@Li) was evaluated under a current density of 0.5 mA cm^−2^, followed by deposition of 1 mA h cm^−2^ after exposure to ambient conditions for 90 min and 4 h [135]. (**g**) An artificial diagram illustrating the LiF@PPF. (**h**) Assessment of the infiltration angle for the separator coated with LiF@PPF and a control sample, in addition to analyzing the electrolyte composition and examining X-ray diffraction patterns of LiF@PPF and PVDF-HFP. Furthermore, conducting Fourier-transform infrared spectroscopy analysis on LiF@PPF and PVDF-HFP. (**i**) The performance of Li symmetrical batteries in different situations [139]. (**j**) Morphology characterization diagram. (**k**) The performance of Li symmetrical batteries in bare Li and LiF-coated Li [134].

Cao et al. [130] fabricated a robust organic-inorganic composite SEI by dissolving LiBF_2_ (C_2_O_4_) (LiD FOB) and azodiisobutyronitrile (AIBN) into poly (ethylene glycol) diacrylate (PEGDA) and stirring to make the precursor solution, then coating it homogeneously onto lithium foil and heating for several hours. As shown in Figure 7a, the deposition behavior of lithium on the modified-lithium electrode was more stable, and the deposition morphology was smoother than that of the bare lithium electrode. The side reactions and dendrite growth can be effectively suppressed by the in situ polymerized artificial SEI, thus improving the chemical and electrochemical stability of LMAs [131]. By leveraging the benefits of this SEI, the Li||LiNi_0.8_Co_0.1_Mn_0.1_O_2_ (NCM811) cells exhibited a sustained capacity exceeding 80 mA h g^−1^ even after 500 cycles at a rate of 0.5 C. Additionally, the Li||Li symmetrical cells demonstrated stable long-term cycling for over 700 h (at a current density of 0.5 mA cm^−2^ and charge capacity of 1 mA h cm^−2^), with a low overpotential of approximately 60 mV (Figure 7b). This engineered SEI offers a straightforward and efficient approach for enhancing the safety and cycling performance in next-generation lithium metal batteries.

An interfacial protective layer with organic (PVDF-HFP) and inorganic (Ag-Li_x_Ag_y_) composites was designed and synthesized on the lithium foil (pa-Li) by Jiang et al. [137]. promoting dendrite-free and highly stable LMAs. As is well-known, Ag possesses excellent lithium wettability and undergoes an alloying process, and it is beneficial to uniform lithium deposition 100,127. More importantly, PVDF-HFP exhibits good ionic conductivity, flexibility, and compatibility, which can compensate for the shortcomings of Ag and Li_x_Ag_y_. Diffusion annealing of the alloy layer, good viscosity of PVDF-HFP, excellent ionic conductivity, and mechanical properties were realized in this composite SEI. Consequently, this stable SEI film can enhance the reaction kinetics of lithium deposition, promote the uniform diffusion of Li^+^, and inhibit the growth of lithium dendrites. Furthermore, the combination of PVDF-HFP organic compounds and Ag-Li_x_Ag_y_ inorganic compounds within the hybrid Li foil protective layer allowed for strong interfacial adherence. This offers a promising approach towards the real-world implementation of lithium metal batteries with enhanced efficiency and an extended lifespan. In the lithium symmetric batteries, the power time can exceed 1000 h at different current densities.

Deng et al. [132] prepared the YF3-doped PAN-based carbon nanofibers (YF3-PAN-CNFs) using a one-step method for lithium metal batteries for the first time. The as-prepared porous CNFs (PCNFs) possess a complex network structure, which promotes infiltration of the liquid electrolyte. As a result, it not only greatly enhances the compatibility between the separator and anode when the coated side is in close proximity to the LMAs but also facilitates the swift transmission of lithium ions and electrons. In addition, the vertical and horizontal twists of the internal pores caused by the disordered arrangement of fibers extend the growth channel of lithium dendrites to a certain extent, avoiding direct piercing of the separator. Moreover, the doped YF3 can provide abundant chemically active sites, and thus the reaction kinetics can be accelerated effectively. As a result, the utilization of a PAN-based CNF-modified separator led to an increase in the cycle time of the symmetrical battery. Moreover, it consistently exhibited a diminished overpotential throughout the 500 h cycles. It is important to mention that YF3-PAN-1 and YF3-PAN-2 (YF3-PAN-1 and YF3-PAN-1 were named according to different experimental methods) demonstrated a voltage distribution pattern similar to that of the PAN-based CNF-modified separator. However, their voltage polarization amplitudes were comparatively smaller than that of the pure PAN-based CNF-modified separator. These findings imply that by applying a layer of CNFs coating on the separator, there is a notable expansion in the growth channel for lithium dendrites and a reduced likelihood of puncturing caused by these dendrites [138].

Li et al. [133] reported that it is efficient and convenient to achieve stable cycling at high current densities by using polyvinyl chloride (PVC) films as artificial layers, in which a SEI rich in LiCl and chlorinated organic components was constructed during cycling (Figure 7c). The XPS results indicated the minimal presence of Li_2_O and Li_2_CO_3_ mineralization products on the surface of the recycled lithium, confirming the stability of this SEI. In addition, dense, uniform, and flat sediments were observed by SEM, indicating that the protective layer was beneficial for inducing the uniform deposition of lithium. With the implementation of this artificial SEI, a consistent and compact lithium deposition structure was established, enabling the attainment of an exceptionally prolonged stable cycling period exceeding 500 h (Figure 7d), even under extremely high current density conditions of 10 mA cm^−2^.

Li et al. [135] proposed a method to construct an organic/inorganic hybrid inter-layer on Li by immersing it in an organic solution containing zinc trifluoromethanesulfonate (Zn(CF_3_SO_3_)_2_) for 1 min (Figure 7e). This specially designed hybrid SEI ensures the high stability of lithium metal when exposed to air for up to 4 h and also prevents the formation of dendrites during cycling of the lithium anode. The DFT calculations revealed that the uniform distribution of LiF and Zn, along with the absorbed -CF_3_ organic groups, effectively reduced the absorption energy of O_2_, H_2_O, and CO_2_ on the surface of lithium, thereby enhancing the air stability of the Li metal anode. The lithium anode, coated with a hybrid SEI consisting of both organic and inorganic components, demonstrates excellent stability over a period of 600 h at a current density of 0.5 mA cm^−2^ when tested in a symmetric cell. Furthermore, it exhibited remarkable durability for up to 500 cycles in a full-cell configuration (Li||LiFePO_4_) after exposure to ambient air for 4 h (Figure 7f).

Yu et al. [139] presented a novel protective film for lithium metal anodes consisting of a combination of soft and rigid materials. The film incorporated LiF nanoparticles that were spatially confined within the aligned channels of the polymer matrix, serving as an artificial inorganic/organic SEI (Figure 7g). By utilizing electrostatic interactions, they successfully prevented the uneven agglomeration of LiF nanoparticles in hierarchical channels made from PVDF-HFP. This porous PVDF-HFP film containing LiF nanoparticles (referred to as LiF@PPF) effectively suppresses undesired side reactions between the electrolyte and the LMA. The hierarchical structure of the LiF@PPF separator had a positive impact on the penetration of the electrolyte, as evidenced by its smaller contact angle compared to that of the blank separator (Figure 7h). This enhanced affinity between the interphase and the electrolyte minimizes the concentration gradients across the artificial SEI, thereby improving the stability of the anode interphase. It is widely acknowledged that β-PVDF possesses excellent dielectric properties and effectively promotes lithium ion homogenization [139,140]. Furthermore, the utilization of PVDF-HFP with a high dielectric constant helps equalize the electric field in close proximity to the lithium metal surface, while incorporating ball-milled LiF nanoparticles aligns and shortens the ion transport pathway within the artificial SEI. The amalgamation of both organic and inorganic components provides adequate durability and robustness. Under a current density of 1 mA/cm2 using a carbonate electrolyte, the Li||Li symmetric battery demonstrated cycling stability for over 900 h (Figure 7i). Additionally, when combined with a LiFePO4 cathode, this anode exhibits commendable performance even under low electrolyte conditions of 9.5 μL mAh^−1^.

Kozen et al. [136] have presented a novel method to enhance the stability of lithium metal anodes. They achieved this by applying a hybrid organic/inorganic artificial SEI directly onto the surface of lithium metal through self-healing electrochemical polymerization (EP) and atomic layer deposition (ALD). This thin, flexible, ionically conductive, and electrically insulating protective layer demonstrated remarkable cycling performance of over 300 cycles at a current density of 1 mA/cm^2^ and more than 110 cycles at a current density of 2 mA/cm^2^. These results surpass the threshold for dendrite growth observed for the unprotected lithium metal. By employing an innovative approach utilizing hybrid organic/inorganic artificial SEIs, they aimed to prevent or minimize dendrite formation on reactive metal anodes such as lithium. This advancement could potentially accelerate the development of “beyond-Li-ion” battery technologies that incorporate LMAs, such as Li-S batteries.

Lang et al. [134] presented a straightforward approach for obtaining LiF coating on LMAs. A chemical method was used to produce the LiF coating by reacting metallic lithium with a solution of polyvinylidene fluoride (PVDF)-dimethyl formamide (DMF) in situ (Figure 7j). The introduction of this chemically and mechanically stable artificial SEI significantly enhanced the cycling performance of the lithium anode compared to using a bare lithium anode in symmetrical cells, even at different current densities. Remarkably, they achieved stable cycling over 300 plating/stripping cycles with LiF-coated lithium electrodes at a high current density of 3 mA cm^−2^ (Figure 7k). Additionally, the application of the LiF coating effectively inhibited dendrite formation and reduced undesired reactions between metallic lithium and carbonate-based electrolytes. Consequently, this cost-effective and simple technique has great potential for advancing future applications in next-generation lithium metal batteries.

Lithium protection layers that combine organic and inorganic materials possess advantageous characteristics such as exceptional mechanical strength and high conductivity for lithium-ion movement [141]. To optimize the utilization of this hybrid SEI design, it is essential to establish guidelines for its structure and composition. Furthermore, for the successful commercialization of LMBs, it is imperative to develop a simple, cost-efficient, environment-friendly, and scalable method for creating a dependable lithium protection layer [142]. This is also one of the main research directions for inorganic/organic SEIs.

### 4.4. Alloy Interphase Layer

The stress change in the negative electrode material of the lithium metal battery during the process of lithium is significantly affected by the rate of lithium, which determines the speed of lithium ion embedding in the material and subsequently affects the distribution and magnitude of the internal stress of the material [143]. Under the reaction control mechanism, the lithium reaction leads to the formation of an obvious lithium-poor and lithium-rich phase boundary inside the material [144]. As the reaction proceeds at the core–shell boundary, the reacted core generates a tensile circumference direction stress on the shell surface, which may cause cracks on the material surface and affect the cyclic stability of the battery. In contrast, the process of lithium under diffusion-controlled mechanisms, such as those observed in amorphous silicon and magnesium, where the diffusion of lithium ions forms a progressive concentration gradient without forming sharp phase boundaries, usually produces circumferent compressive stresses at the surface, a mechanism that helps maintain the structural integrity of the material. Therefore, depending on the lithium dynamics of the negative electrode material, the stress caused by diffusion can be significantly different, thereby determining the mechanical stability of the material. In recent years, based on these principles, researchers have devoted themselves to developing new negative electrode materials and design strategies (i.e., alloyed SEI) to optimize the stress distribution during the lithium process, improve the safety and cycle stability of the battery, and use advanced characterization techniques and computational simulations to better understand the mechanical behavior of the material and predict and control the stress changes during the battery charging and discharging processes. This has promoted the development of LMB technology to achieve higher energy density and longer cycle life.

**Figure 8:**
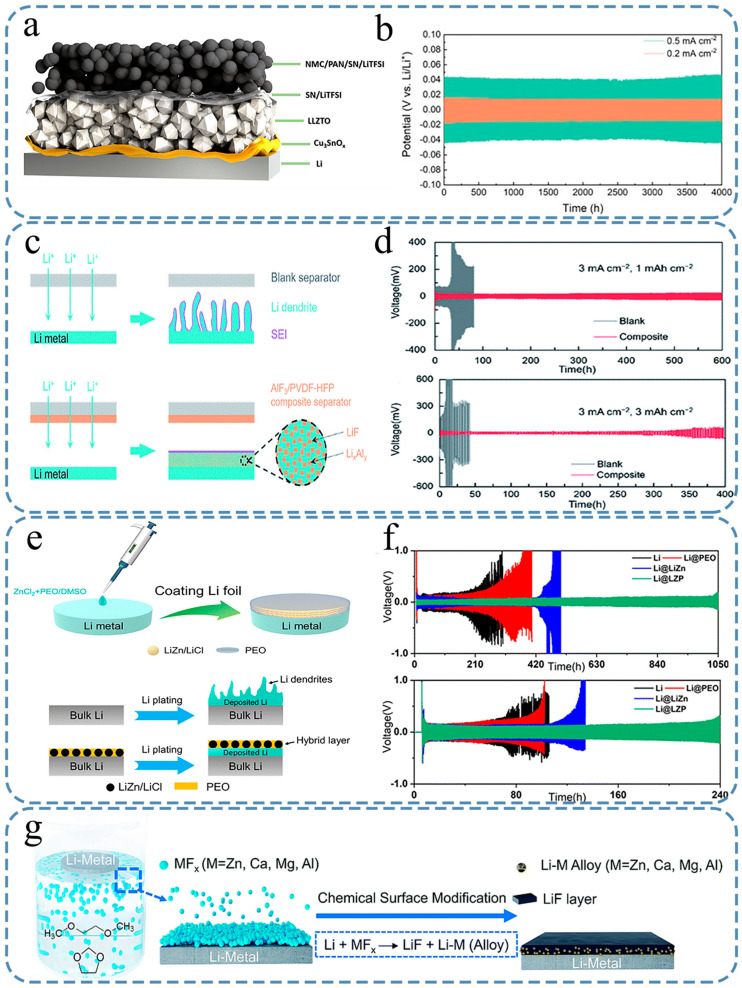
Effects of different alloy components on lithium metal batteries and SEI synthesis diagram. (**a**) Schematic diagram of the microstructure and material of the whole battery. (**b**) The performance of the Li symmetrical batteries [145]. (**c**) Schematic of Li deposition with a blank separator, lithium dendrites form after cycling, and with the AlF_3_/PVDF-HFP composite separator. (**d**) Electrochemical analysis of Li||Li symmetrical batteries with separators composed of AlF_3_/PVDF-HFP composite and blank materials [147]. (**e**) Preparation diagram and morphology evolution diagram. (**f**) The performance of the Li symmetrical batteries in different circumstances [153]. (**g**) The formation of the SEI is depicted in a schematic manner through ex situ chemical modification [160].

Cui et al. [145] developed a rapid heating system and used it to design an ultrathin coating layer of Cu_z_Sn_y_O_x_ (6/5 < z/y < 3) on the surface of the garnet electrolyte, which is capable of eliminating the interphase resistance between the garnet-type electrolyte and LMAs completely, leading to a dendrite-free operation of LMBs at high current densities and low temperatures for a long period (Figure 8a). After their approach effectively eliminated interfacial resistance, the stability of the modified garnet electrolyte solely depended on its intrinsic properties. This resulted in a remarkably high critical current density of 15.2 mA cm^−2^ at 60 °C (Figure 8b). Their experimental findings showed an all-solid-state full cell incorporating a modified garnet electrolyte and a LiNi_0.8_Mn_0.1_Co_0.1_O_2_ cathode, which exhibited an impressive capacity retention rate of 94% after 1000 stable cycles at room temperature.

A novel approach to creating an artificial SEI with dual functionality has been proposed and developed by Ma et al. [146]. This method utilizes surface chemistry techniques to simultaneously incorporate both passivation and active site effects into the layer. The resulting LiF component effectively stabilizes the anode/electrolyte interphase, leading to an improved cycling performance over time. Additionally, the presence of a Li-Mg solid solution alloy promotes the efficient transmission of lithium ions and facilitates low-energy deposition of lithium in a uniform manner (MF-Li). Taking advantage of these benefits, the Li||Li cell featuring the modified anode exhibited a reduced nucleation overpotential of only 2.3 mV and an exceptionally long cycling lifespan exceeding 2000 h at a current density of 1 mA cm^−2^. Additionally, the Li||LiFePO_4_ full battery maintained, it maintains an impressive capacity retention rate of 84.6% even after 300 cycles at a rate of 1 C.

Wang et al. [147] presented a simple method for applying a protective layer onto the surface of the LMA by directly positioning a PVDF-HFP/AlF_3_ modified Celgard separator on the top. As illustrated in Figure 8c, the LiF-rich SEI on the surface of the lithium anode effectively regulates the behavior of lithium deposition and successfully inhibits the formation of lithium dendrites [148,149]. This electrochemically deposited Li-Al alloy plays a crucial role in forming an in situ SEI that can modify the way lithium is deposited [150,151]. Additionally, this solid solution exhibits excellent ductility similar to that of pure metal, which distinguishes it from brittle intermetallics. Consequently, SEIs composed of intermetallic compounds are prone to cracking or damage during cycling. Consequently, the formation of an in situ SEI protective layer on the LMA plays a crucial role in stabilizing the interphase between the lithium metal and electrolyte, thereby enabling an extended lifespan and preventing dendrite formation on LMAs. Owing to the improved stability of the interphase, the Li||Li symmetrical battery demonstrated a consistent polarization voltage for approximately 600 h in comparison to the blank cell’s duration of 45 h (Figure 8d). The Li||LiFePO_4_ cell, equipped with the composite separator, exhibits an impressive capacity retention rate of 78.3% after undergoing 300 cycles at a rate of 3C. This signifies a notable enhancement in the longevity of high-energy-density Li metal batteries during the cycling tests.

Zhao [152] developed a protective layer called interfacial compatible protective layer with interpenetrating Li-Sn alloy across the PVDF-HFP interphase (ISPI) and compared the effects of different experimental schemes. This layer was applied on the lithium surface to seamlessly integrate the organic and inorganic components in the interphase and to create a smooth interphase between the SEI and metallic lithium. Unlike traditional double-layered or uniform protective layers with distinct joint interphases, the ISPI demonstrated improved mechanical stability. Additionally, it ensured even deposition of lithium underneath, even when operating at a high capacity of 5 mAh/cm^2^. By utilizing the ISPI-Li anode, which is compatible with this interphase, the Li||Li symmetric cells demonstrated stable performance even at a remarkably high current density of 20 mA cm^−2^. These cells maintained a capacity of 1 mAh cm^−2^ for over 1000 cycles. Additionally, when combined with either LiFePO_4_ or LiNi_0.8_Co_0.1_Mn_0.1_O_2_ (NCM811) cathodes, the full cells equipped with ISPI-Li anodes exhibited exceptional cycling performance. This study introduces a significant concept for designing an artificial SEI that seamlessly integrates with lithium metal anodes and reveals the crucial role of interfacial compatibility in achieving a highly reversible lithium anode suitable for practical usage in LMBs.

Wang et al. [153] developed a protective layer for the LMA that combines a Li-Zn alloy and PEO polymer. This hybrid layer effectively prevents the formation of dendrites on the surface of the LMA (Figure 8e). The use of the Li-Zn alloy facilitates fast transport of lithium ions, which are uniformly distributed in the PEO matrix to ensure even electronic and ion flux distribution. Additionally, the flexible PEO network helps mitigate volume changes during cycling. As a result, this synergistic approach allows for the deposition of lithium underneath the hybrid film, leading to significantly improved cycling stability compared with using only pristine LMAs. A composite protective layer enables a Li||Li symmetric battery to achieve a cycling duration of over 1000 h at a current density of 1 mA cm^−2^, while maintaining a fixed capacity of 1 mA h cm^−2^ (Figure 8f). Furthermore, when combined with a high-areal-capacity LiFePO_4_ cathode (2.45 mA h cm^−2^), the full cell demonstrated exceptional cycling performance.

Ma et al. [154] prepared a unique hybrid SEI film by introducing an aluminum coordination compound (Al(MMP)_3_), which effectively inhibited the side reactions and growth of lithium dendrites, as well as adjusted the deposition stability of lithium. This hybrid film contains a flexible fluoropolymer (TGD) and Al(MMP)_3_, which can be converted in situ into a dual alloy of a Li_x_Al-Li_x_P hybrid interphase. This SEI has a high ionic conductivity while enhancing the flexible and rigid hybrid interphase, effectively avoiding the accumulation of inactive lithium. After incorporating the hybrid anode, the LMB demonstrated remarkable electrochemical longevity of over 2000 h at a current density of 1 mA cm^−2^. Additionally, when combined with a Ni-rich cathode, it exhibits exceptional capacity preservation and consistent coulombic efficiency.

All in all, there are many studies on this series of Li–M alloy layers [155] (such as Li-Mg [146], Li-Al [147,156], Li-In [157], Li-B [158], Li-Zn [153,159] and Li-Sn [146,149,155]). By comparing the fluoride formation effects of different alloys, Li et al. [160] found that a fluorinated alloy-type interphase layer can effectively inhibit the growth of undesirable lithium dendrites and improve the performance of lithium metal anodes (Figure 8g). These alloys serve as a pathway for rapid diffusion of lithium ions, preventing their reduction on the lithium surface and effectively inhibiting the growth of lithium dendrites. However, inherent dendrite formation is still inevitable. Additionally, not all alloys contribute to enhancing the coulombic efficiency of the lithium anode, which is associated with the alloy resistance. For instance, incorporating the SnI_2_ additive resulted in decreased coulombic efficiency due to the elevated resistance of Li-Sn alloy layers [161]. Hence, by combining the advantages of the aforementioned approaches, superior protection for the lithium anode can be achieved.

The latest research progress is summarized in Table 1. According to the above experimental investigations and research, recent research directions can be summarized as organic SEIs, inorganic SEIs, organic/inorganic composite SEI films, and alloy SEIs. In the application of organic SEI films, the main focus is on polymer research. The main concerns for inorganic SEIs are LiF and LiCl. However, whether LiF actually contributes to the formation of an effective SEI has been questioned by other studies and needs further verification [83]. Many studies have shown that SEIs containing LiF have excellent ion transport capabilities and enhance the conductivity of lithium ions, but there are still few studies on whether LiF really plays a role, and further verification is needed. For organic/inorganic composite SEI, its performance exceeds that of a single inorganic or organic layer. This SEI dynamically combines the respective advantages of inorganic and organic protective layers while avoiding their disadvantages. This composite structure not only improves the mechanical properties of the interphase but also enhances the inhibition effect on lithium dendrites, thus significantly improving the cycle performance and stability of the battery. The key research mechanism is the transport effect of lithium ions and insulation performance of the electronic tunnel. For the alloy SEI, the SEI lithium mainly focuses on the in situ reaction to form the alloy layer. Compared with other types of SEI films, the production process is relatively simple, and the main performance advantage lies in the high mechanical strength achieved by utilizing defects in the alloy layer. Some metals have certain solubility in lithium metal and react easily to form solid solutions. The surface layers of these solid solutions have a high degree of matching with the crystal structure of lithium metal, which provides a buffer effect for subsequent lithium deposition and effectively reduces the nucleation barrier. It also provides a fast lithium-ion transport channel, which can effectively guide the uniform deposition of lithium metal. The alloy protective layer not only maintains good mechanical strength but also mitigates volume changes during the cycle [162]. By reasonably combining the LiF-rich and LiN_x_O_y_-rich SEIs, it can effectively promote the transmission efficiency of lithium, promote its stable deposition, reduce the growth of lithium dendrites, and ensure the safety performance of LMBs.

Overall, an SEI prepared using a variety of composite materials will have a better effect and be more conducive to the development of artificial SEIs.

## 5. Conclusions and Prospects

**Figure 9:**
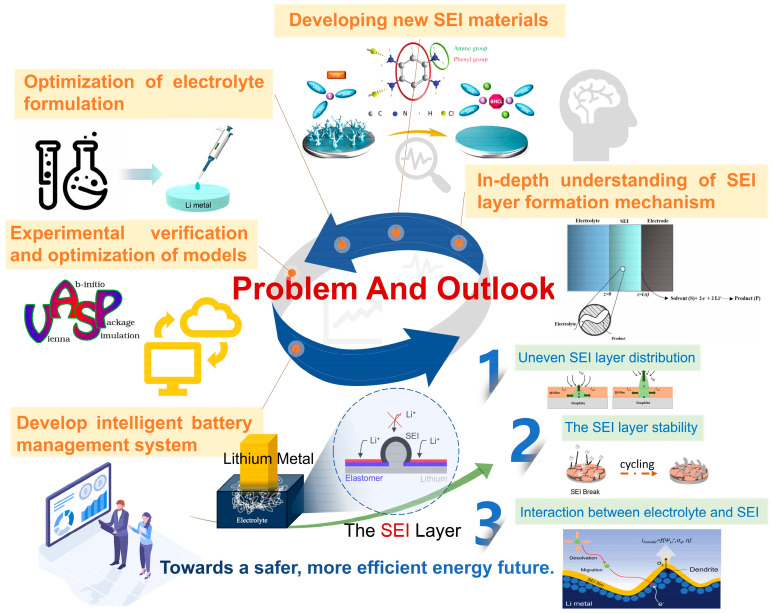
Problem and outlook of the SEI in lithium metal battery.

Although the research of artificial SEIs for lithium metal anodes has made good progress, in the face of the current demand for high-energy-density batteries, the SEI research on lithium metal batteries still faces the following challenges: (a) The uneven distribution of SEIs may lead to uneven lithium deposition, increasing the risk of dendrite growth. (b) During long-term cycles, the SEIs may break or degrade, requiring continuous maintenance and repair. (c) The composition and additives of the electrolytes have an important impact on the formation and stability of the SEIs, which is not yet fully understood. In addition, although the existing mathematical models can predict the battery performance, their applicability and accuracy in real battery systems still need to be verified. As illustrated in Figure 9, The future research on the SEIs can be focused on the following aspects: (a) In-depth understanding of the SEI formation mechanism: reveal the detailed process and influencing factors of SEI formation by advanced characterization techniques and computational simulation. In order to deeply understand the formation mechanism of SEI, we can start from two aspects: experimental characterization and computational simulation. Experimentally, in situ electrochemical microscopy, XPS [163], and cryo-electron microscopy were used to observe the formation and evolution of SEI in real time and analyze its composition, structure, and distribution. In the aspect of computational simulation, MD simulation and DFT [163,164] calculations are used to study the interaction between lithium ions and electrolyte molecules, the reaction process, and the growth mechanism of SEI from the atomic scale and to predict its stability and composition. (b) Explore novel inorganic, organic, and composite SEI materials to further improve the performance and safety of LMBs. Inorganic materials such as LiF [165] and metal phosphates can enhance the mechanical stability and chemical inertness of SEI and inhibit the growth of lithium dendrites. Organic materials such as fluorine-containing organics and polymers can improve the flexibility and ionic conductivity of SEI and reduce the transmission impedance. Composite materials can comprehensively improve the performance of SEI by combining the advantages of inorganic and organic materials, such as multi-layer structure design or nanocomposite technology. (c) Optimize the electrolyte additives and solvents to achieve a more stable SEI. In terms of additives, such as composite additives of fluoroethylene carbonate (FEC) and BSTFA, they can balance the organic and inorganic components of SEI, inhibit the growth of lithium dendrites, and improve battery performance [166]. In terms of solvents, selecting a suitable solvent system, such as a solvent containing siloxane, can form an SEI rich in organic components, reduce the transmission and desolvation impedance, and improve the low-temperature performance of the battery. At the same time, the comprehensive performance of SEI can be further improved by optimizing the combination of additives and solvents. (d) By combining the mathematical models with the experimental results, continuously optimize the models to better predict the performance of actual battery systems. Firstly, based on the battery characteristics and application scenarios (such as accuracy requirements or real-time requirements), the adaptation model (such as the electrochemical model to analyze the microscopic mechanism and the equivalent circuit model for real-time control) is selected. After evaluating the predictive ability of the model through cross-validation and independent testing, the parameters and structure are iteratively optimized by combining sensitivity analysis and a multi-objective optimization algorithm. The verified model is embedded into the battery management system (BMS) or digital twin platform to realize performance prediction and safety control. Finally, the actual operation data are collected by sensors to form a dynamic closed loop of ’deployment–feedback–calibration’, which continuously improves the applicability and reliability of the model under complex working conditions. (e) Employ the AI and machine learning technology to monitor and manage battery status in real time and extend battery life. Using artificial intelligence and machine learning technology to monitor and manage battery status in real time can accurately evaluate the battery ’s state of charge (SOC), state of health (SOH), and remaining useful life (RUL). By analyzing the trend of battery data mode, predicting faults in advance, dynamically adjusting charging strategies and energy distribution, and preventing harmful behaviors such as overcharging and overdischarging, the battery can always work in the best state, thereby prolonging battery life and improving the safety and reliability of the battery system. In addition, it is undeniable that the use of solid electrolytes is one of the effective strategies to solve the problems existing in lithium metal batteries. Therefore, how to establish a reasonable SEI model of solid electrolyte and explore the artificial SEIs that can be used in solid electrolytes will also become one of the future research hotspots. The chemical stability, interface contact change, and microstructure of solid electrolyte will affect the formation, stability, and ion conductivity of SEI. In the next generation of battery chemistry, the changes in new electrolyte materials, electrode materials, and battery chemical systems will also affect the formation and evolution of SEI. These factors work together to determine the interface stability and battery performance.

In the solid electrolyte system, there are unique challenges in the SEI research of lithium metal batteries: insufficient solid-state interface contact leads to non-uniform growth of SEI and high interface impedance, and the dynamic evolution mechanism (such as chemical compatibility between solid electrolyte and lithium metal, stress-induced ion transport heterogeneity) is not clear; there is a lack of multi-physical field coupling models for solid-state systems (such as interface stress-electrochemical synergistic effect, the effect of solid electrolyte grain boundaries on SEI composition) and cross-scale in situ characterization techniques (such as atomic-scale dynamic observation of solid electrolyte/lithium interface); in addition, the solid-state composite SEI design (such as the synergistic passivation of inorganic solid electrolyte and artificial interface layer) faces problems such as poor mechanical compatibility (interfacial peeling under cyclic volume deformation), high cost of large-scale preparation and insufficient long-term stability verification, which restrict the practical process of all-solid-state lithium metal batteries.

After considering the impact of SEI performance, the pros and cons of economic trade-offs cannot be ignored. The development of new electrolyte materials or additives to optimize SEI performance may increase the cost of raw materials. For example, the use of new materials such as solid electrolytes or ionic liquids is usually more expensive than traditional liquid electrolytes, which affects the overall manufacturing cost of the battery. In order to form a stable SEI, it may be necessary to improve the manufacturing process of the battery, such as adding pretreatment steps or using special coating technology, which will increase the complexity and cost of the production process. By optimizing the SEI, the cycle stability of the battery can be significantly improved, and the capacity loss during each charge and discharge process can be reduced. For example, in lithium-ion batteries, stable SEI can inhibit the growth of lithium dendrites and reduce the corrosion and pulverization of electrode materials, thereby prolonging the cycle life of the battery and reducing the replacement cost. Although optimizing the SEI may increase the initial investment cost, by significantly extending the battery life, the cost can be shared over the entire life cycle of the battery, thereby improving the economy. For example, in electric vehicles, battery packs using high-performance SEI technology, although more expensive to manufacture, can reduce the frequency of battery replacement and reduce the long-term use cost of users due to their longer cycle life and better performance retention. Different SEI optimization strategies have different cost-effectiveness ratios. For example, the use of some new additives may be less costly and can effectively improve SEI performance, while the development of new solid electrolyte materials may require higher investment and face the challenges of technological maturity and large-scale production. In practical applications, it is necessary to select the appropriate SEI optimization technology according to specific needs and economic conditions to achieve the best balance between cost and performance.

In summary, the research of SEIs is crucial for improving the performance of LMBs. Through continuous material innovation and theoretical development, we can expect to achieve safer, more stable, and more efficient lithium metal battery technology in the future. With a deeper understanding of the SEI and the application of new materials and modeling techniques, the commercialization and large-scale application of LMBs will no longer be far away.

## Figures and Tables

**Table 1:** The performance and electrochemical situation of SEI with different structure composition (symmetrical batteries). The symbol ‘√’ means that the SEI has certain specified properties (under this column, such as, ionconductivity, mechanical strength and high stability).

Type of SEI Film	Ionconductivity	Mechanical Strength	High Stability	Electrochemical Performance	Testing Condition	Ref.
LiF-rich	√	√		over 1700 h	1 mA cm^−2^/1 mAh cm^−2^	[107]
				over 1400 h	2 mA cm^−2^/2 mAh cm^−2^	
FCF			√	over 500 h	1 mA cm^−2^/1 mAh cm^−2^	[109]
				over 300 h	3 mA cm^−2^/1 mAh cm^−2^	
LiF/LiCl/LiIn	√		√	over 1000 h	1 mA cm^−2^/1 mAh cm^−2^	[110]
LiCl-rich	√		√	over 2500 h	1 mAhcm^−2^/1 mAh cm^−2^	[111]
				over 1000 h	5 mA cm^−2^/5 mAh cm^−2^	
LiBFEP-enriched	√			over 600 h	0.1 mA cm^−2^/1 mAh cm^−2^	[112]
Li_2_O-rich			√	over 400 h	0.5 mA cm^−2^/1 mAh cm^−2^	[113]
				over 300 h	1 mA cm^−2^/1 mAh cm^−2^	
PVDF/PAMPSLi			√	over 350 h	2 mA cm^−2^/1 mAh cm^−2^	[121]
				over 150 h	5 mA cm^−2^/1 mAh cm^−2^	
Carboxylate			√	over 1000 h	0.5 mA cm^−2^/1 mAh cm^−2^	[122]
PEO			√	over 500 h	1 mA cm^−2^/1 mAh cm^−2^	[123]
NCL	√	√	√	over 1000 h	1 mA cm^−2^/1 mAh cm^−2^	[124]
				over 1100 h	1 mA cm^−2^/3 mAh cm^−2^	
Terpolymer			√	over 800 h	0.5 mA cm^−2^/1 mAh cm^−2^	[125]
AIBN-PEGDA			√	over 700 h	0.5 mA cm^−2^/1 mAh cm^−2^	[130]
PVDF-HFP/Ag-Li_x_Ag_y_	√	√		over 1000 h	0.25 mA cm^−2^/0.5 mA h cm^−2^	[137]
				over 1400 h	1 mA cm^−2^/1 mAh cm^−2^	
YF3-PAN-CNFs	√		√	over 500 h	1 mA cm^−2^/1 mAh cm^−2^	[132]
LiCl-rich (PVC)			√	over 500 h	1 mA cm^−2^/1 mAh cm^−2^	[133]
Zn(CF_3_SO_3_)_2_	√		√	over 700 h	1 mA cm^−2^/2 mAh cm^−2^	[135]
LiF@PPF		√	√	over 900 h	1 mA cm^−2^/1 mAh cm^−2^	[139]
EP-ALD		√		over 550 h	1 mA cm^−2^/1 mAh cm^−2^	[136]
				over 220 h	2 mA cm^−2^/2 mAh cm^−2^	
PVDF/DMF/LiF		√		over 600 h	1 mA cm^−2^/1 mAh cm^−2^	[134]
				over 200 h	3 mA cm^−2^/1 mAh cm^−2^	
Cu_z_Sn_y_O_x_	√		√	over 1600 h	4 mA cm^−2^/2 mA cm^−2^	[145]
Li-Mg	√		√	over 2000 h	1 mA cm^−2^/1 mAh cm^−2^	[146]
PVDF-HFP/AlF_3_		√	√	over 600 h	3 mA cm^−2^/1 mAh cm^−2^	[147]
				over 400 h	3 mA cm^−2^/3 mAh cm^−2^	
ISPI		√		over 700 h	3 mA cm^−2^/1 mAh cm^−2^	[152]
				over 1000 h	1 mA cm^−2^/5 mAh cm^−2^	
Li-Zn/PEO	√		√	over 1000 h	1 mA cm^−2^/1 mAh cm^−2^	[153]
TGD/Al(MMP)_3_	√	√	√	over 2000 h	1 mA cm^−2^/1 mAh cm^−2^	[154]

## Abbreviations

The following abbreviations are used in this manuscript:

SEI	solid electrolyte interphase
LMBs	lithium metal batteries
LMA	lithium metal anode
CES	Consumer Electronics Show
Si	silicon
LUMO	lowest unoccupied molecular orbital
EC	ethylene carbonate
DMC	dimethyl carbonate
FTIR	Fourier transform infrared spectrometer
XPS	X-ray photoelectron spectroscopy
TEM	transmission electron microscopy
EQCM	electrochemical quartz crystal microbalance
ToF-SIMS	time-of-flight secondary ion mass spectrometry
DFT	density functional theory
MD	molecular dynamics
PFM	phase-field models
MC	Monte Carlo
EC	ethylene carbonate
LiEC	lithium ethyl carbonate
Li_2_BDC	dilithium butylene dicarbonate
LiPC	lithium phthalocyanine
UFF	universal force field
COMPASS	Condensed-phase Optimized Molecular Potentials for Atomistic Simulation Studies
ReaxFF	Reactive Force Field
VC	vinylene carbonate
FEC	fluoroethylene carbonate
AFM	atomic force microscope
SIMS	secondary ion mass spectrometry
FIB	focused ion beam
cryo-TEM	cryogenic transmission electron microscopy
kMC	kinetic Monte Carlo
LiFSI	lithium bis(fluorosulfonyl)imide
F5DEE	1-(2,2-difluoroethoxy)-2-(2,2,2-trifluoroethoxy)ethane
LIBs	lithium-ion batteries
ASBs	all-solid-state batteries
AI	artificial intelligence
LiPF_6_	lithium hexafluorophosphate
ROCO_2_Li	lithium semicarbonates
FES	finite element simulations
DOL	1,3-dioxolane
BHCL	1,2,4,5-benzenetetramine with high amine content
DFEB	2, 3-difluoroethoxy-benzene
LHCEs	localized high-concentration electrolytes
SPAN	sulfurized polyacrylonitrile
CE	coulombic efficiency
PAMPSLi	PVDF-lithium poly (acrylamide-2-methyl-1-propane-sulfonate)
HNT	halloysite nanotubes
PEO	polyethylene oxide
LiPAA	Li polyacrylic acid
AIBN	azodiisobutyronitrile
PEGDA	poly (ethylene glycol) diacrylate
YF3-PAN-CNFs	YF3-doped PAN-based carbon nanofibers
PCNFs	as-prepared porous CNFs
PVC	polyvinyl chloride
EP	electrochemical polymerization
ALD	atomic layer deposition
PVDF	polyvinylidene fluoride
DMF	dimethyl formamide
Al(MMP)_3_	aluminum coordination compound
